# Egr-1 Induces a Profibrotic Injury/Repair Gene Program Associated with Systemic Sclerosis

**DOI:** 10.1371/journal.pone.0023082

**Published:** 2011-09-13

**Authors:** Swati Bhattacharyya, Jennifer L. Sargent, Pan Du, Simon Lin, Warren G. Tourtellotte, Kazuhiko Takehara, Michael L. Whitfield, John Varga

**Affiliations:** 1 Division of Rheumatology, Feinberg School of Medicine, Northwestern University, Chicago, Illinois, United States of America; 2 Department of Genetics, Dartmouth Medical School, Hanover, New Hampshire, United States of America; 3 Northwestern University Clinical and Translational Sciences Institute, Chicago, Illinois, United States of America; 4 Departments of Pathology and Neurology, Feinberg School of Medicine, Northwestern University, Chicago, Illinois, United States of America; 5 Department of Dermatology, Kanazawa University, Kanazawa, Japan; Universidade Federal do Rio de Janeiro, Brazil

## Abstract

Transforming growth factor-ß (TGF-ß) signaling is implicated in the pathogenesis of fibrosis in scleroderma or systemic sclerosis (SSc), but the precise mechanisms are poorly understood. The immediate-early gene Egr-1 is an inducible transcription factor with key roles in mediating fibrotic TGF-ß responses. To elucidate Egr-1 function in SSc-associated fibrosis, we examined change in gene expression induced by Egr-1 in human fibroblasts at the genome-wide level. Using microarray expression analysis, we derived a fibroblast “Egr-1-responsive gene signature” comprising over 600 genes involved in cell proliferation, TGF-ß signaling, wound healing, extracellular matrix synthesis and vascular development. The experimentally derived “Egr-1-responsive gene signature” was then evaluated in an expression microarray dataset comprising skin biopsies from 27 patients with localized and systemic forms of scleroderma and six healthy controls. We found that the “Egr-1 responsive gene signature” was substantially enriched in the “diffuse-proliferation” subset comprising exclusively of patients with diffuse cutaneous SSc (dcSSc) of skin biopsies. A number of Egr-1-regulated genes was also associated with the “inflammatory” intrinsic subset. Only a minority of Egr-1-regulated genes was concordantly regulated by TGF-ß. These results indicate that Egr-1 induces a distinct profibrotic/wound healing gene expression program in fibroblasts that is associated with skin biopsies from SSc patients with diffuse cutaneous disease. These observations suggest that targeting Egr-1 expression or activity might be a novel therapeutic strategy to control fibrosis in specific SSc subsets.

## Introduction

Systemic sclerosis (SSc) is a complex disease of unknown cause with variable clinical manifestation, substantial molecular heterogeneity and unpredictable course [1]. While vascular injury and autoimmunity are prominent in early disease, fibrosis ultimately develops in most patients, and is responsible for organ failure and a poor prognosis. Transforming growth factor-ß (TGF-ß) serves as a potent stimulus for collagen gene transcription, myofibroblast differentiation, and other fibrotic responses [2]. Since TGF-ß expression and activity are deregulated in SSc, TGF-ß is considered a major factor contributing to pathogenesis [3]. Precise delineation of transcription factors and cofactors that comprise the fibroblast-specific intracellular TGF-ß signal transduction pathways is indispensable for developing effective anti-fibrotic therapies [4].

The immediate-early gene product Egr-1 is a zinc finger transcription factor induced by environmental stress, developmental signals, cytokines, growth factors, hypoxia and oxidative stress [5]. We recently demonstrated that TGF-ß stimulates Egr-1 mRNA and protein expression in normal fibroblasts in a rapid and transient manner [6], [7]. Moreover, TGF-ß-induced stimulation of collagen gene expression in these cells was found to be mediated by Egr-1, which was on its own capable of inducing COL1A2 transactivation, indicating a vital functional role in profibrotic TGF-ß-responses. Indeed, Egr-1 expression was found to be elevated in lesional skin tissues from mice with bleomycin-induced scleroderma, as well as in skin and lung biopsies from patients with diffuse cutaneous SSc [7]. Together, these observations indicate a novel functional role for Egr-1 in the regulation of connective tissue homeostasis, and suggest that abnormal sustained Egr-1 expression might contribute to progression of fibrosis in SSc.

To better understand the implications of Egr-1 activity in the context of fibrosis, we examined gene regulation by Egr-1 in primary human skin fibroblasts at the genome-wide level. Transcriptional profiling by DNA microarray analysis identified 647 genes whose expression in fibroblasts was significantly changed by Egr-1. These genes are involved in cell proliferation, TGF-ß signaling, wound healing, extracellular matrix synthesis and vascular development. Querying a microarray-based gene expression dataset from skin biopsies from patients with localized and systemic forms of scleroderma and healthy controls showed that the “Egr-1-regulated gene signature” was most prominent in skin biopsies clustering within the “diffuse-proliferation” intrinsic subsets of SSc biopsies, but some of the genes were also associated with “inflammatory” subset. These results indicate that Egr-1 exerts potent regulatory effects on a substantial number of fibroblast genes that are functionally implicated in matrix remodeling, tissue repair and pathological fibrosis. The Egr-1-regulated gene signature only partially overlapped with TGF-ß-regulated genes in fibroblasts, and was most prominent in skin biopsies from patients with diffuse cutaneous SSc, implicating Egr-1-mediated fibroblast activation in these patients. These findings point to a previously unrecognized role for Egr-1 in the pathogenesis of SSc, and raise the possibility that blocking excessive Egr-1 signaling might be a potential therapeutic strategy to control fibrosis.

## Materials and Methods

### Cell culture and reagents

Cultures of human primary fibroblasts were established by explantation from neonatal foreskin and studied at early (<8) passage [8]. Cultures were maintained in modified Eagle's medium (EMEM) supplemented with 10% fetal calf serum (FCS) (Gibco BRL, Grand Island, NY), 1% vitamin solutions, and 2 mM L-glutamine. All other tissue culture reagents were from Biowhittaker (Walkersville, MD). In some experiments, fibroblasts were incubated with TGF-ß1 (PeproTech, Rocky Hill, NJ) for up to 48 h.

### Immunohistochemistry

Skin biopsies from the lesional forearm from six patients with SSc and three healthy adults were obtained under protocols approved by the Institutional Review Boards for Human Studies at Northwestern University or Kanazawa University. Four µm thick paraffin-embedded skin sections were deparaffinized, rehydrated and immersed in TBS-T buffer (Tris-buffered saline- 0.1%Tween 20) followed by target retrieval solution (DAKO, Carpinteria, CA). Following incubation of the slides with primary antibodies against Egr-1 (Santa Cruz, 1∶100 dilution), cartilage oligomeric matrix protein (COMP) (Accurate Chemical & Scientific; Westbury, MY; dilution 1∶50), E2F7 (Abcam; Cambridge, MA; dilution; 1∶200) or GDF6 (Epitomics; Burlingame, CA; dilution 1∶50), bound antibodies were detected using DAKO Envision+System. After counterstaining with hematoxylin, sections were mounted with Permount (Fisher Scientific, Pittsburgh, PA) and viewed under a Nuance Multiple Spectra microscope.

### Infection with adenovirus

Adenoviral recombinants containing active Egr1 that does not contain the NAB inhibitory domain mutant of Egr-1 (Ad-Egr-1m) [9], and Ad-EGFP expressing the green fluorescent protein (GFP) were amplified and used for infecting human fibroblasts. At early confluence, fibroblasts in serum-free media were infected with adenovirus (100 MOI). Following 24–48 h incubation, cells were harvested, total RNA or whole cell lysates were isolated, and processed for microarray, real-time quantitative PCR or Western analysis.

### Microarray procedures

For genome-wide analysis of the Egr-1 response, confluent fibroblasts in 100 mm dishes were cultured in to serum-free media immediately prior to adenovirus infection. Total RNA was isolated from two independent fibroblast cultures for each time point (24 and 48 h) using RNeasy mini kit (Qiagen, Valencia, CA). The integrity of RNA was ascertained by an Agilent Bioanalyzer (Santa Clara, CA). cDNA was labeled using an Ambion labeling kit (Ambion) and was hybridized to Illumina Human Ref-6 version 2 Expression Microarray Chips (Illumina, San Diego, CA).

### Data Analysis

Raw signal intensities for each probe were obtained using Illumina Beadstudio data analysis software and imported to the Bioconductor lumi package for data transformation and normalization [10], [11]. The data were preprocessed using variance stabilization transformation method [12] followed by quantile normalization. Probes with all samples “absent” (near or below background levels) were filtered. The rest of the probes were used for further analysis. Differential analysis was performed using Bioconductor limma package [13]. The variance used in the t-score calculation was corrected by an empirical Bayesian method for better estimation with a small sample size [13]. To control the effects of multiple testing and reduce the false positive rate (FDR), stringent statistical criteria were used to identify differentially expressed genes with p values less than 0.01 and fold-induction>2 fold [14]. Comparisons of data for Egr-1m versus control and TGF-ß versus control were performed separately by Ingenuity Pathway Analysis (Ingenuity, Mountain View, CA).

The microarray dataset of skin biopsies from 27 patients with various forms of scleroderma and healthy controls, was downloaded from the UNC Microarray Database and is also available from GEO (Accession GSE9285). This dataset has been described in detail [15].

### Real-time quantitative PCR

Total RNA (50 ng) was reverse-transcribed to cDNA using Reverse Transcription System (Promega, Madison, WI) [7]. The products were amplified using SYBR Green PCR Master Mix (Applied Biosytems, Foster City, CA) on the Applied Biosystems 7500 Prism Sequence Detection System. The primers used are shown in Table 1.

**Table 1:** Oligonucleotides used as primers for Real-Time Quantitative PCR.

TIMP3	Forward	5′CCTTTGGCACTCTGGTCTACA3′
	Reverse	5′GTCCCACCTCTCCACAAAGTT3′
COMP	Forward	5′ AGC ACC GGC CCC AAG T 3′
	Reverse	5′ GGT TGT GCC AAG ACC ACG TT3′
KIAA1199	Forward	5′ GTG AGC CAC ACG AGC TTC AG3′
	Reverse	5′ GGA TGG TCG CCA CATAGT TGA3′
MMP3	Forward	5′ ACA AAG GAT ACA ACA GGG ACC AA3′
	Reverse	5′ TAG AGT GGG TAC ATC AAA GCT TCA GT3′
PDGFC	Forward	5′ CAA GGA ACA GAA CGG AGT ACA AGA3′
	Reverse	5′ CCA TAC ATT TTC CTCTAC TGC TAC TAA TCT3′
BGN	Forward	5′ TGTTCCCTCCATCTCTCCGAACCTG 3′
	Reverse	5′ GACCGCTGTCCCTGGGGTTTTG3′
FN	Forward	5′ GCCTGGTACAGAATATGTAGTG3′
	Reverse	5′ ATCCCAGCTGATCAGTAGGCTGGTG3′
PLOD2	Forward	5′ CAGAAGGAACAGCTGGGAGTG3′
	Reverse	5′ GTGGTGACTGCGAGGGCTT3′

### Western Analysis

At the end of each experiment, cultures were harvested, whole cell lysates were isolated and equal amounts of proteins (20–50 µg/lane) were subjected to electrophoresis in 4–15% SDS polyacrylamide gradient gels [7]. Proteins were transferred to Immobilon-P membranes (Millipore, Billerica, MA) and membranes were probed sequentially with primary antibodies specific for Egr-1 (C19), actin (C2) (both from Santa Cruz Biotechnology, Santa Cruz, CA) or Type I collagen (Southern Biotech, Birmingham, AL). Membranes were then incubated with appropriate secondary antibodies and subjected to enhanced chemiluminescence detection using ECL reagent (Amersham-Pharmacia, Piscataway, NJ).

### Statistical Methods

Statistical significance for real-time qPCR results was determined using the unpaired Student's t-test. A p value<0.05 was considered significant.

### Data access

Microarray data have been deposited at GEO (http://www.ncbi.nlm.nih.gov/geo/; accession no. GSE27165 (provisional). All data are MIAME compliant.

## Results

### Adenoviral-Egr-1infection results in Egr-1 overexpression in skin fibroblasts

**Figure 1:**
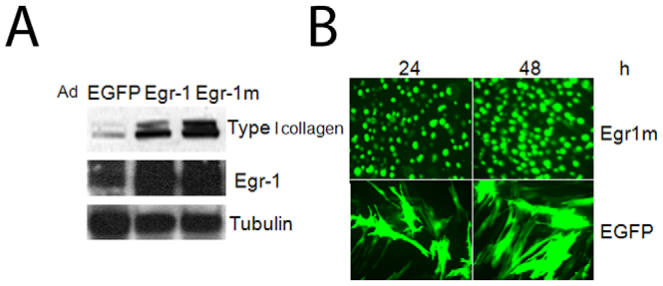
Adenovirus-mediated Egr-1 expression in human skin fibroblasts. Confluent dermal fibroblasts were infected with Ad-EGFP, Egr-1 or Egr-1m (100 MOI) for 48 h. **A.** Cultures were harvested and whole cell lysates were subjected to Western analysis. Representative immunoblots. **B.** Fibroblasts were examined by fluorescence microscopy. Representative image. (Original magnification ×400).

To define optimal experimental condition, we first examined the effects of Egr-1 or a transcriptionally active Egr1 that is refractory to it endogenous inhibitor Nab2 [16]. Normal skin fibroblasts were infected in parallel with indicated concentrations of Ad Egr-1 or AdEgr-1m or the control vector Ad-EGFP and incubated for various periods up to 48 h. Ectopic Egr-1 expression in infected fibroblasts resulted in a ∼6-fold increase in the cellular abundance of Egr-1 (Fig. 1A), which is comparable to the magnitude of increase in Egr-1 induced by TGF-ß [6]. Accumulation of Egr-1 in infected fibroblasts was accompanied by marked stimulation of Type I collagen synthesis. As expected, Egr-1m elicited a more robust stimulatory response than wildtype Egr-1, since resistance to Nab2 in this Egr-1 mutant permitted unfettered target gene stimulation. Immunofluorescence analysis confirmed maximal Egr-1 expression at 48 h in fibroblasts infected with 100 MOI Ad-EGFP (Fig. 1B). No effect on cell viability was detected under these conditions.

### Identification of Egr-1 target genes by Illumina Microarray analysis

**Figure 2:**
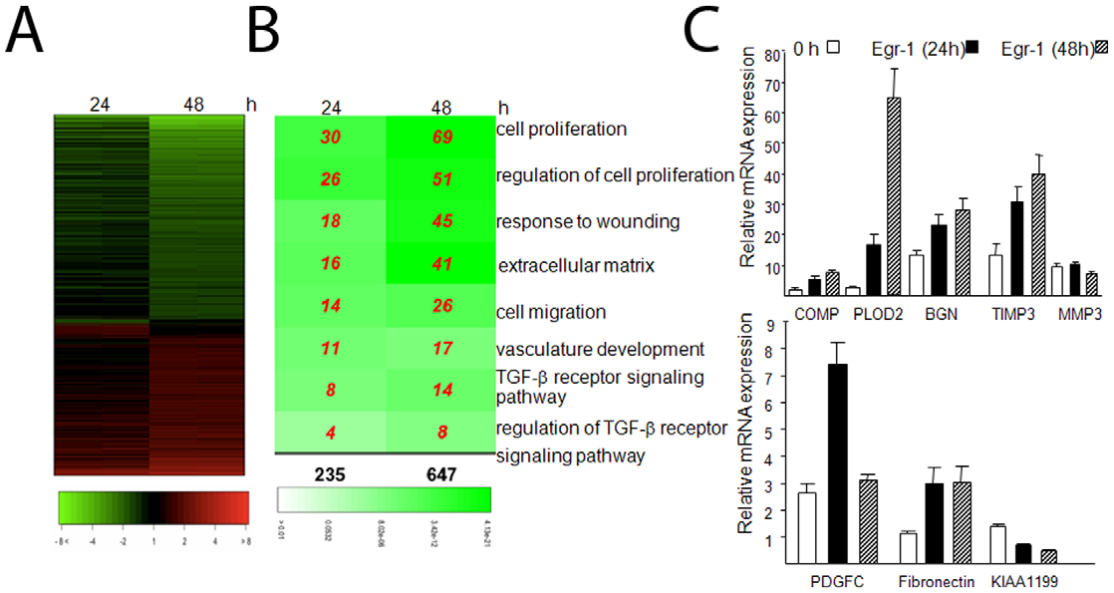
Egr-1-induced transcriptional responses in skin fibroblasts. Dermal fibroblasts were infected with Ad-EGFP or Ad-Egr-1m (100 MOI). At the end of 24 or 48 h incubation, total RNA was isolated and subjected to genome-wide transcriptional analysis using Illumina Microarray chips (A,B) or real-time qPCR (C). **A.** Heatmap of differentially expressed genes (FDR<0.01 and >2- fold-change) (48 h). The color represents the fold-change of Egr-1 in comparison with the average of control sample (red = increased, green = decreased). Each row represents a probe and each column represents one sample. Genes with similar changes in expression pattern compared to the control are clustered together for 24 and 48 h. **B.** Comparing a subset of biological processes significantly enriched (p<0.001) with Egr-1-regulated genes at 24 and 48 h. The number in the plot indicates the differentially expressed genes belonging to individual GO categories (row) at corresponding time point (column). The total number of genes at each time point (column) is shown below the Table. The background color represents the statistical significance of a particular biological process overrepresented in the differential gene list as estimated by Hyper-geometric test. C. Real-time qPCR. Results, normalized with GAPDH mRNA, are the means ± S.D. of triplicate determinations from a representative experiment.

To evaluate gene expression changes induced by Egr-1 at the genome-wide level, confluent skin fibroblasts were infected with Ad-Egr-1m for up to 48 h and total RNA processed for microarray analysis. To reduce the false positive rate, probes with all samples “Absent” were filtered for further analysis, leaving 12915 probes. Gene expression in Egr1m-infected fibroblasts was compared to that of Ad-EGFP-infected fibroblasts at each time point, duplicate samples for each time point showed similar changes in gene expression patterns. Probes that showed a >2-fold change in Egr-1m-infected fibroblasts compared to control-infected cultures at each time point were selected for further analysis. At 24 h 235 genes showed significantly altered expression (p<10^−5^; FDR<0.01), with 109 genes showing increased expression and 126 genes decreased expression (Fig. 2A). At 48 h, 647 genes demonstrated significantly altered expression, with 261 genes increased and 386 genes decreased. This 647-gene cohort was defined as the fibroblast “Egr-1-responsive gene signature”. The top Egr-1-regulated genes at 24 h and 48 h (p<0.001) are shown in Tables S1 and S2.

### Genome-wide responses to Egr-1

To identify biological processes enriched significantly (p<0.001; Hyper-geometric test) with Egr-1-regulated genes, GO analysis was performed (Fig. 2B). The list of enriched biological processes includes cell proliferation, cell migration, extracellular matrix synthesis, wound healing, vascular development and TGF-ß receptor signaling, all of which are known to be implicated in wound healing, tissue remodeling and fibrosis. A time-dependent increase in the number of genes associated with each biological process was observed. GO analysis confirmed that Egr-1 induced a time-dependent ECM gene expression program, with the number of ECM genes increasing >2.5-fold (from 16 to 41) between 24 and 48 h. Genes in this group include those coding for multiple collagens (COL4A1, COL4A2, COL11A1, COL7A1, COL10A1), biglycan, fibronectin, COMP, procollagen-lysine, 2-oxoglutarate 5-dioxygenase2 (PLOD2), and tissue inhibitor of matrix metalloproteinase 3 (TIMP3). The observed pattern of Egr-1-induced changes in gene expression therefore is consistent with the notion that persistent Egr-1 signaling in fibroblasts induces tissue remodeling, wound healing and fibrogenesis gene program.

### Validation of the “Egr-1-responsive gene signature” in fibroblasts

Real-time qPCR was used to validate Egr-1-induced changes in fibroblasts ECM gene expression. For this purpose, RNA from the same samples that were used for DNA microarrays were subjected to further analysis by real-time qPCR. The changes in the expression of eight Egr-1-induced ECM genes were compared to the expression from the DNA microarrays. In each case, expression changes determined by the real-time qPCR showed the same direction of change as was seen in microarray analysis (Fig. 2C, and data not shown).

### Overlap of the Egr-1-regulated gene signature and the TGF-ß-regulated gene signature

**Figure 3:**
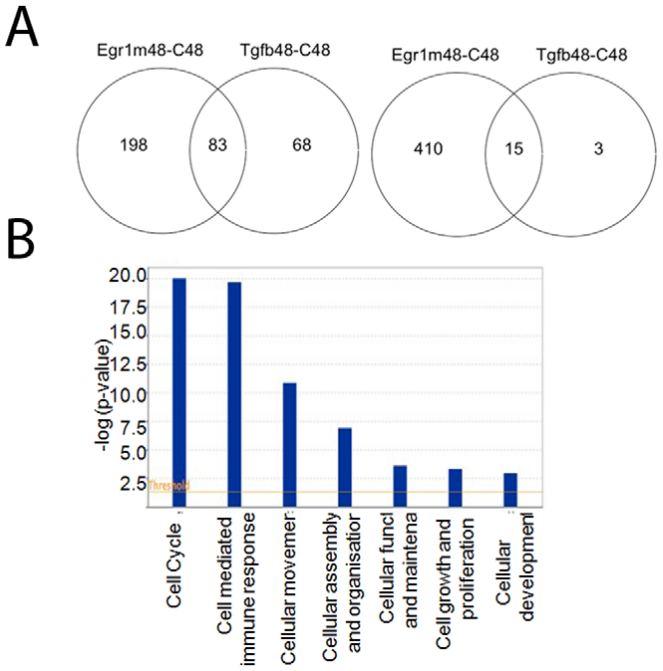
Genes regulated by both TGF-ß and AdEgr-1. Skin fibroblasts were infected with Ad-EGFP or incubated with TGF-ß in parallel for 48 h. Total RNA was subjected to microarray analysis using Illumina chips. **A.** Venn diagrams of the genes regulated by TGF-ß and Egr-1. Left, up-regulated genes; right, down-regulated genes. **B.** Ingenuity Pathway Analysis showing canonical signaling pathways enriched with 98 fibroblast genes regulated by both Egr-1 and TGF-ß. The Y axis shows the pathway enrichment.

Because TGF-ß induces a robust time-dependent up-regulation of Egr-1 along with numerous profibrotic genes [17], [18], and since Egr-1 itself plays a role in mediating TGF-ß-mediated fibrotic responses [6], [7], we sought to compare response induced by Egr-1 and by TGF-ß in normal fibroblasts at the genome-wide level using microarrays. Analysis of the data showed that while at 48 h of incubation, TGF-ß and Egr-1 modulated the expression of 158 and 647 genes, respectively, only 98 of these genes were regulated simultaneously by both TGF-ß and Egr-1, with 83/98 genes showing concordantly increased, and 15/98 genes showing concordantly decreased, expression (Fig. 3A, and Table S3). Ingenuity Pathway Analysis showed that these 98 concordantly-regulated genes are implicated in cell cycle regulation, cell proliferation, cellular assembly and organization, cellular function and maintenance, cellular development and movement, and cell-mediated immune responses (Fig. 3B).

### The fibroblasts Egr-1-responsive gene signature in scleroderma skin biopsies

**Figure 4:**
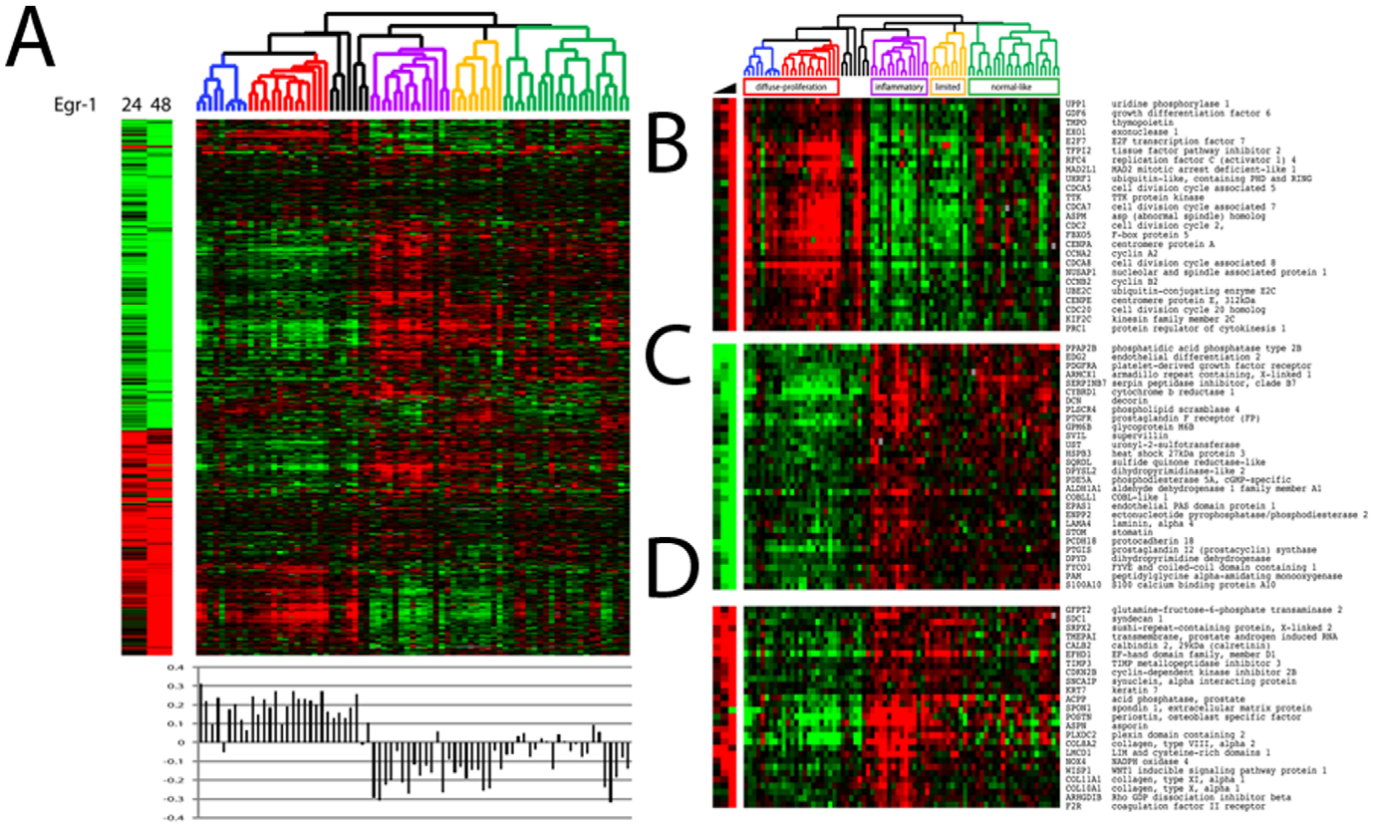
Expression of the Egr-1-responsive gene signature in SSc skin biopsies. **A.** Egr-1-responsive genes are aligned with the gene expression data from dcSSc and healthy control skin biopsies. The samples were clustered using the 647 genes that comprise Egr-1-responsive gene signature. Dendrogram shows clear differences in skin biopsies. The left branch of the dendogram (highlighted in red and blue), comprises solely dcSSc biopsies clustering with the diffuse-proliferation intrinsic subsets (diffuse 1 and diffuse 2). The right branch includes remaining dcSSc samples, as well as lcSSc, localized scleroderma and all healthy controls. Quantitation of Egr-1 signaling in each biopsy by Pearson correlation is shown below the heatmap. **B.** Genes showing high expression in dcSSc skin biopsies and in Egr-1-expressing fibroblasts. **C.** Genes associated with the inflammatory and normal-like intrinsic subsets, or low expression in proliferation subset and Egr-1-expressing fibroblasts. **D.** Genes showing low expression in the proliferation subset but high expression in Egr-1-expressing fibroblasts and inflammatory subset.

To determine the clinical implications of activated Egr-1 signaling, we examined the fibroblast “Egr-1-responsive gene signature” in scleroderma skin biopsies. For this purpose, a genome-wide microarray dataset comprising biopsies of lesional and non-lesional skin from patients with various forms of scleroderma (dcSSc, lcSSc and localized) and healthy controls was [15]. Data for each gene identified above as Egr-1-responsive were extracted from the microarray dataset, and the samples were ordered according to the intrinsic gene clustering described previously [15]. A heatmap of the 75-biopsy microarray dataset of scleroderma and healthy skin biopsies was generated (Fig. 4A), with the Egr-1-regulated gene signature shown to the left. In each sample, the level of enrichment with the “Egr-1-responsive signature” was quantified by calculating the Pearson correlation coefficients between the Egr-1 centroid and the gene expression data (Fig. 4A, lower panel). As shown in Figure 4, biopsies clustering in the diffuse-proliferation intrinsic subsets (blue and red dendograms) showed significant enrichment with “Egr-1-responsive gene signature” (average Pearson correlation 0.1837±0.0772) compared to all other samples (average Pearson correlation −0.1000±0.1238, p<1×10^−18^). Interestingly, these intrinsic subsets were found previously to be significantly enriched with the “TGF-ß-responsive gene signature” [18], providing evidence for the relationship between TGF-ß and Egr-1 signaling in skin fibrosis. Assessment of the clinical features indicated that these SSc patient subsets had higher Rodnan skin scores, and greater frequency of lung involvement [18].

The expression of “Egr1-responsive signature” genes across each of the intrinsic subsets is presented in Table S4. Genes whose expression is suppressed by Egr-1 in fibroblasts and that show reduced expression in a particular biopsy subset are shown in green; and genes that are stimulated by Egr-1 in fibroblasts and show enhanced expression in the biopsies are shown in red. Analysis of these data indicate that 53% of Egr-1-regulated genes showed a concordant direction of change (increase or decrease) in the diffuse-proliferative subset of skin biopsies; in contrast, 14% of Egr-1-regulated genes showed a concordant pattern of change in expression in the fibroblasts and skin biopsies clustering with the inflammatory intrinsic subset (S4).

Further analysis of the expression of Egr-1-regulated genes in the microarray dataset showed that while most of the Egr-1-regulated genes were strongly associated with the diffuse-proliferative intrinsic biopsies subsets, a group of genes including TIMP3, Nox4, syndecan, collagen X and collagen XI and WISP1 was prominent in the “inflammatory' and “limited” subsets of skin biopsies, and was not significantly changed in the “diffuse proliferation” subsets (Fig. 4B–D).

Of the 98 genes coordinately regulated in fibroblasts by both Egr-1 and TGF-ß, 73 were found to be present in the scleroderma biopsy microarray dataset. The “diffuse-proliferation” intrinsic subsets were substantially enriched with these genes which are involved in cell cycle regulation and cell proliferation (Fig. S1 and data not shown).

### Expression of Egr-1 and target genes in SSc skin biopsies

**Figure 5:**
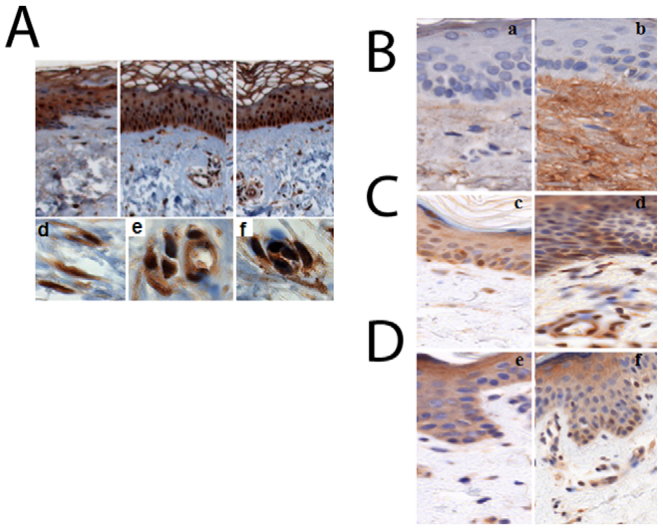
Expression of the Egr-1 and its target genes in SSc skin biopsies. Immunohistochemistry. Skin biopsies from SSc patients and healthy controls were immunostained with antibodies. Representative images. **A.** Egr-1. a healthy control, b–f SSc. (a–c original magnification, ×100; d–f original magnification, ×400). **B–D.** COMP, E2F7 and GDF6. a,c,e healthy controls; b,d,f SSc.(Original magnification ×400).

The expression of Egr-1 in SSc was examined by immunohistochemistry. For this purpose, skin biopsies from patients with early dcSSc (<1 year) and age-matched healthy controls were studied in parallel. The results showed that in contrast to control biopsies that had little or no detectable Egr-1 in the dermis, in SSc biopsies a significant proportion of fibroblastic cells, as well as some vascular cells, showed distinct Egr-1 immunostaining (Fig. 5A). In the epidermis, SSc samples and healthy controls showed similar Egr-1 levels. The expression of selected Egr-1-regulated genes was next examined. Immunohistochemistry showed that COMP, an Egr-1-regulated ECM protein known to be induced by TGF-β, was strongly expressed throughout the dermis in SSc biopsies, but was sparse in control biopsies (Fig. 5A). E2F7, a cell cycle regulator that is known as a target of TGF-ß (Sargent et al, JID), and is also regulated by Egr-1, was found to be elevated in some fibroblasts in SSc skin biopsies but not in control (Fig. 5B). E2F7 immunostaining was also seen in some vascular cells and in keratinocytes in the SSc biopsies. Growth differentiation factor-6 (GDF6), a member of the TGF-ß superfamily that is also regulated by both TGF-β and Egr-1, was up-regulated in the basal epidermis, dermal fibroblasts and in vascular cells in SSc skin biopsies compared to control biopsies (Fig. 5C).

## Discussion

We previously identified Egr-1 as a novel mediator of TGF-ß-induced collagen stimulation that is up-regulated in skin and lung biopsies of patients with SSc [7]. In this study, we investigated genome-wide Egr-1 transcriptional responses in normal fibroblasts by DNA microarray analysis. Egr-1 was shown to modulate the expression of 647 genes that define the fibroblast “Egr-1-responsive gene signature”. This signature comprises genes involved in cell proliferation and migration, ECM synthesis, wound healing, and vascular development and shows only a partial overlaps with TGF-ß-regulated gene signature. Importantly, this gene signature is highly expressed in skin biopsies from subsets of dcSSc patients, but is not found in patients with lSSc, or morphea, or in healthy controls.

In immortalized vascular endothelial cells, ectopic Egr-1 induced the transcription of more than 300 genes [16]. These genes are involved in transcriptional regulations, signaling, cell cycle, cytokines and ECM biosynthesis. In normal skin fibroblasts, Egr-1 altered the expression of a partially overlapping set of 647 genes involved in cell proliferation, migration, ECM synthesis, wound healing, vascular development and TGF-ß receptor signaling, suggesting cell type-specific differences in Egr-1 transcriptional responses.

Rapid and generally transient Egr-1 induction is essential for orchestrating acute tissue responses to various forms of injury [19]. Emerging evidence implicates Egr-1 in physiological wound healing on one hand, and pathological tissue repair on the other [20]. We found that the ECM genes collagen, biglycan, fibronectin, COMP, and PLOD2 were up-regulated by Egr-1. Since Egr-1 is persistently overexpressed in SSc skin and lung biopsies from SSc patients, it might drive unchecked target gene activation resulting in fibrosis. In particular, our results show a strong “Egr-1-responsive gene signature” expression in the skin biopsies clustering with the ‘diffuse-proliferation’ SSc subset but not with biopsies from patients with limited SSc or morphea, or healthy controls. This intrinsic SSc subset was shown previously to be associated with a higher skin scores and incidence of lung involvement [18].

Scleroderma is characterized by substantial patient-to-patient heterogeneity in presentation, autoantibody profiles, clinical outcome and molecular signatures [2]. DNA microarray analysis of gene expression in skin biopsies provides evidence for distinct subsets of scleroderma distinguishable by their gene expression patterns [15]. The present results raise the possibility that dcSSc patients expressing the “Egr-1 responsive gene signature” represent a distinct molecular subset whose disease is driven by Egr-1, and who might therefore benefit from interventions specifically targeting Egr-1. Several drugs in current clinical use have potent effects on Egr-1 expression. These include mycophenolate mofetil [21], cyclosporine [22], simvastatin [23], imatinib mesylate [24] and insulin-sensitizing PPARγ ligands such as rosiglitazone [25], [26]. In summary, the present results demonstrate that persistent Egr-1 expression in normal fibroblasts induces substantial genome-wide change in gene expression, with robust up-regulation of wound healing and fibrogenic gene expression program. A subset of skin biopsies from patients with dcSSc, but not other forms of scleroderma, show evidence of robust Egr-1-dependent gene activation. In view of the aberrant Egr-1 or its signature gene expression seen in SSc and other forms of pathological fibrosis, these results suggest that sustained Egr-1 signaling could be implicated in SSc fibrogenesis, and blocking Egr-1 signaling pathway may be of therapeutic benefit in controlling the progression of fibrosis.

## Supporting Information

Figure S1 The expression of the Egr1/TGFβ overlapping genes in the SSc skin dataset.
(PPTX)

Table S1 Top gene information of Egr1m24-C24 and TGF-β 24-C24 (p-value<0.001) and fold-change larger than two.
(DOCX)

Table S2 Top gene information of Egr1m48-C48 and TGF-β 48-C48 (p-value<0.001) and fold-change larger than two.
(DOCX)

Table S3 The expression of the Egr1/TGFβ overlapping genes.
(XLS)

Table S4 Expression of the Egr-1 signature across the subsets.
(XLSX)
