# Supplementary figures and images for: Egr-1 Induces a Profibrotic Injury/Repair Gene Program Associated with Systemic Sclerosis
Source: PLoS One. 2011 Sep 13;6(9):e23082. doi: 10.1371/journal.pone.0023082 (PMC3172216; doi:10.1371/journal.pone.0023082)

## Slide 1
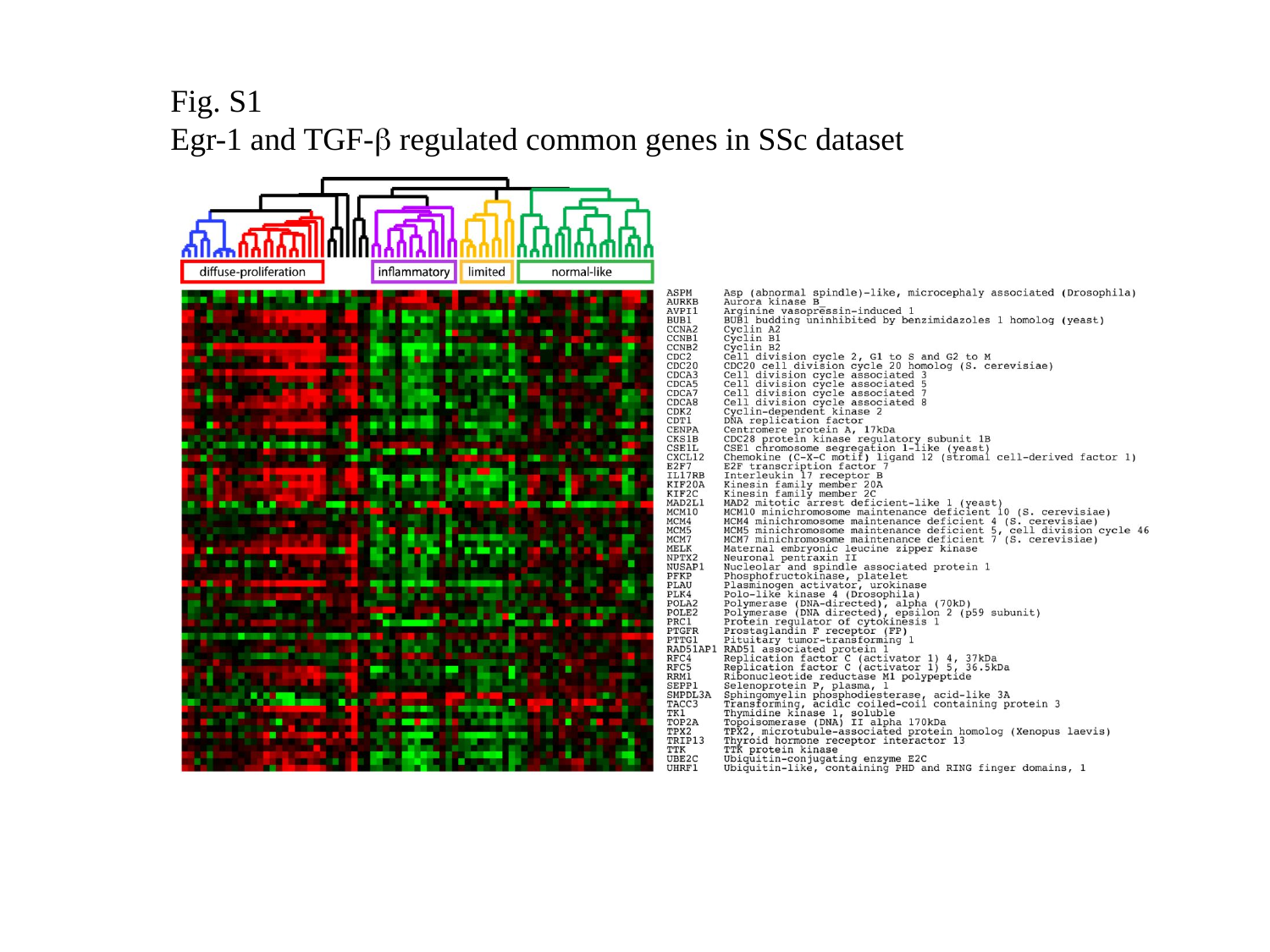

Fig. S1
Egr-1 and TGF-b regulated common genes in SSc dataset

Supplement: Figure S1 — The expression of the Egr1/TGFβ overlapping genes in the SSc skin dataset. (PPTX) [file pone.0023082.s001.pptx]
