# Supplementary material for: Egr-1 Induces a Profibrotic Injury/Repair Gene Program Associated with Systemic Sclerosis
Source: PLoS One. 2011 Sep 13;6(9):e23082. doi: 10.1371/journal.pone.0023082 (PMC3172216; doi:10.1371/journal.pone.0023082)
Supplement: Table S1 — Top gene information of Egr1m24-C24 and TGF-β 24-C24 (p-value<0.001) and fold-change larger than two. (DOCX) [file pone.0023082.s002.docx]

**Table S1**

####

**Table S3**

Genes upregulated by both Egr-1 m and TGF-β at 48 h

| nuID | Entrez Id | Gene Symbol | Gene Name | Fold Change_Egr1m48-C48 | Fold Change_Tgfb48-C48 | p.value_Egr1m48-C48 | p.value_Tgfb48-C48 | FDR_Egr1m48-C48 | FDR_Tgfb48-C48 |
| --- | --- | --- | --- | --- | --- | --- | --- | --- | --- |
| 6zhu2h0pSegTuSV6Ls | 5214 | PFKP | phosphofructokinase, platelet | 6.1306 | 2.7078 | 1.63E-08 | 5.11E-07 | 6.58E-06 | 0.00013459 |
| Wg57u3taRVrKXj6v3I | 6790 | AURKA | aurora kinase A | 5.2286 | 4.2199 | 1.15E-07 | 2.55E-07 | 1.18E-05 | 9.58E-05 |
| HClLklAJJdNRQpNZ4I | 991 | CDC20 | cell division cycle 20 homolog (S. cerevisiae) | 4.4782 | 3.7359 | 5.45E-07 | 1.14E-06 | 2.72E-05 | 0.00017529 |
| xX7dOhfRSFxdAkUSTk | 890 | CCNA2 | cyclin A2 | 4.3486 | 3.2794 | 2.73E-08 | 9.31E-08 | 6.64E-06 | 5.65E-05 |
| 6V04FQT4y1fgRE55Yk | 11065 | UBE2C | ubiquitin-conjugating enzyme E2C | 4.0306 | 4.5637 | 1.15E-07 | 7.03E-08 | 1.18E-05 | 4.54E-05 |
| clyu1_J4FkeCB37XR4 | 113130 | CDCA5 | cell division cycle associated 5 | 3.6159 | 4.1004 | 2.46E-08 | 1.44E-08 | 6.64E-06 | 3.09E-05 |
| ZqR1Qw4LulILoN.Eoc | 983 | CDC2 | cell division cycle 2, G1 to S and G2 to M | 3.5726 | 3.1711 | 2.63E-08 | 4.64E-08 | 6.64E-06 | 4.41E-05 |
| Q9G111OeKZDEWferSg | 55839 | CENPN | centromere protein N | 3.4842 | 2.3402 | 7.29E-07 | 6.49E-06 | 3.20E-05 | 0.00047114 |
| TqCLahRL88UPeKC1l8 | 4085 | MAD2L1 | MAD2 mitotic arrest deficient-like 1 (yeast) | 3.4798 | 3.3798 | 9.29E-07 | 1.06E-06 | 3.75E-05 | 0.00016964 |
| irp0SIKOkjrklBDnhY | 51659 | GINS2 | GINS complex subunit 2 (Psf2 homolog) | 3.4537 | 3.5929 | 3.34E-08 | 2.79E-08 | 6.87E-06 | 4.41E-05 |
| lazqZV5PnqO.eVGv_8 | 9319 | TRIP13 | thyroid hormone receptor interactor 13 | 3.4308 | 3.5342 | 5.10E-06 | 4.45E-06 | 9.91E-05 | 0.00037936 |
| HLhL9JyhgSiu0h54JY | 64151 | NCAPG | non-SMC condensin I complex, subunit G | 3.3755 | 3.6438 | 6.11E-06 | 4.32E-06 | 0.00011032 | 0.00037717 |
| WJeRGp_rq9f6II7p6A | 25966 | C2CD2 | C2 calcium-dependent domain containing 2 | 3.2936 | 2.3052 | 1.71E-07 | 1.31E-06 | 1.45E-05 | 0.00018028 |
| iTtHHqiu9edyfUY4pk | 9055 | PRC1 | protein regulator of cytokinesis 1 | 3.214 | 3.5349 | 1.46E-06 | 9.30E-07 | 4.65E-05 | 0.00016019 |
| BiXUgqHpd5URfh9LP0 | 81620 | CDT1 | chromatin licensing and DNA replication factor 1 | 3.1413 | 2.5284 | 3.86E-08 | 1.30E-07 | 7.13E-06 | 6.15E-05 |
| 0nu9VB3veZGASze.ik | 55165 | CEP55 | centrosomal protein 55kDa | 3.1302 | 3.3459 | 2.99E-06 | 2.17E-06 | 7.47E-05 | 0.00023498 |
| WV5e7U7SH_J414MMCU | 51514 | DTL | denticleless homolog (Drosophila) | 3.127 | 4.6164 | 7.17E-07 | 1.33E-07 | 3.18E-05 | 6.15E-05 |
| uUlHrBT33HezlTitD4 | 9585 | KIF20B | kinesin family member 20B | 3.1013 | 2.5835 | 3.50E-08 | 9.65E-08 | 7.01E-06 | 5.65E-05 |
| 3KgVb_bmVB1QukAKD4 | 9768 | KIAA0101 | KIAA0101 | 3.0047 | 3.2045 | 3.70E-07 | 2.67E-07 | 2.21E-05 | 9.58E-05 |
| WdiMSf.ks66vr.rsE0 | 6491 | STIL | SCL/TAL1 interrupting locus | 2.9891 | 2.9465 | 1.22E-05 | 1.31E-05 | 0.00017587 | 0.00069 |
| rSTjV0ngdAJbQIX5dU | 9133 | CCNB2 | cyclin B2 | 2.9747 | 2.6857 | 1.06E-06 | 1.86E-06 | 3.95E-05 | 0.00022267 |
| TuUn0AFY7rqzSikmnw | 9833 | MELK | maternal embryonic leucine zipper kinase | 2.9496 | 2.4097 | 3.06E-08 | 1.01E-07 | 6.87E-06 | 5.65E-05 |
| 9kpQkqQVRFfhMAk34I | 84798 | C19orf48 | chromosome 19 open reading frame 48 | 2.8997 | 2.4221 | 4.24E-06 | 1.21E-05 | 8.99E-05 | 0.00065713 |
| QI4q05JB0nbQ0HU47o | 5984 | RFC4 | replication factor C (activator 1) 4, 37kDa | 2.8953 | 2.5307 | 5.67E-07 | 1.23E-06 | 2.79E-05 | 0.00017836 |
| NLdHHg5Rffj0UrcB5I | 9787 | DLGAP5 | discs, large (Drosophila) homolog-associated protein 5 | 2.8731 | 2.6711 | 7.43E-06 | 1.11E-05 | 0.0001251 | 0.00063768 |
| 3p3cVJyBF9SEfnoSc0 | 23234 | DNAJC9 | DnaJ (Hsp40) homolog, subfamily C, member 9 | 2.8685 | 3.0809 | 3.13E-06 | 2.15E-06 | 7.70E-05 | 0.00023498 |
| rUvfXhFo6ufqBb_JfM | 29128 | UHRF1 | ubiquitin-like with PHD and ring finger domains 1 | 2.7976 | 4.3396 | 2.48E-05 | 3.34E-06 | 0.00027822 | 0.0003223 |
| xp6k.U0zIXeV9Isl0U | 1058 | CENPA | centromere protein A | 2.7506 | 2.0897 | 1.69E-07 | 1.04E-06 | 1.45E-05 | 0.00016801 |
| lekrQ59WhGjR.7ePUk | 4176 | MCM7 | minichromosome maintenance complex component 7 | 2.7441 | 3.8081 | 2.58E-07 | 5.12E-08 | 1.74E-05 | 4.41E-05 |
| iEf0jCK7_eo6CBxek4 | 55010 | C12orf48 | chromosome 12 open reading frame 48 | 2.7052 | 2.3972 | 2.57E-06 | 5.36E-06 | 6.80E-05 | 0.00042487 |
| TkSOS3qk6.u56H906U | 4173 | MCM4 | minichromosome maintenance complex component 4 | 2.6904 | 3.2303 | 1.79E-07 | 6.74E-08 | 1.50E-05 | 4.54E-05 |
| x.Sd_F7Vd6eXeLeDdU | 7153 | TOP2A | topoisomerase (DNA) II alpha 170kDa | 2.6829 | 3.5325 | 5.23E-06 | 1.29E-06 | 0.00010032 | 0.00018009 |
| xp.pD390lxFdH77o04 | 29028 | ATAD2 | ATPase family, AAA domain containing 2 | 2.6753 | 2.5642 | 4.38E-06 | 5.63E-06 | 9.13E-05 | 0.00043259 |
| xij3cugh0gPpG7ngoE | 51053 | GMNN | geminin, DNA replication inhibitor | 2.654 | 2.5961 | 1.66E-05 | 1.89E-05 | 0.00021409 | 0.00083423 |
| Teq_16d6oiIaJfyxJw | 11004 | KIF2C | kinesin family member 2C | 2.632 | 2.0915 | 1.73E-07 | 8.21E-07 | 1.46E-05 | 0.00015586 |
| NjXGdGm9V.HQN6l.6Q | 10112 | KIF20A | kinesin family member 20A | 2.6298 | 2.2495 | 3.43E-06 | 9.31E-06 | 8.02E-05 | 0.00059086 |
| 9pNIw5RQKITNHnnqH4 | 3037 | HAS2 | hyaluronan synthase 2 | 2.6048 | 3.087 | 0.0016713 | 0.0007293 | 0.0061462 | 0.0083649 |
| NRBiFzZZNtCuhcC9Vk | 9212 | AURKB | aurora kinase B | 2.5991 | 2.8375 | 2.85E-06 | 1.73E-06 | 7.27E-05 | 0.00021077 |
| ipUX0u7gHYLn60Xg_8 | 220042 | C11orf82 | chromosome 11 open reading frame 82 | 2.5944 | 2.6644 | 1.35E-06 | 1.15E-06 | 4.44E-05 | 0.00017529 |
| rr5wo6ngnqt6F9DQJc | 9232 | PTTG1 | pituitary tumor-transforming 1 | 2.5882 | 2.1194 | 2.27E-07 | 8.78E-07 | 1.66E-05 | 0.00015852 |
| Th3rxI_343fo3XSN.o | 55872 | PBK | PDZ binding kinase | 2.5585 | 2.9374 | 1.11E-05 | 5.13E-06 | 0.00016754 | 0.0004133 |
| NlJfXQg5SEuhIgk5CE | 29089 | UBE2T | ubiquitin-conjugating enzyme E2T (putative) | 2.5557 | 2.2654 | 3.17E-06 | 6.91E-06 | 7.75E-05 | 0.00048815 |
| ccp.nyzTtTv19F0uic | 51203 | NUSAP1 | nucleolar and spindle associated protein 1 | 2.5482 | 3.1185 | 2.32E-06 | 7.59E-07 | 6.37E-05 | 0.00014858 |
| BepQfvreD3XB1P_Ank | 7272 | TTK | TTK protein kinase | 2.543 | 2.5426 | 2.21E-07 | 2.21E-07 | 1.64E-05 | 8.93E-05 |
| 0ofRQkgMVokZTUQrnk | 4174 | MCM5 | minichromosome maintenance complex component 5 | 2.5389 | 2.5472 | 3.44E-07 | 3.37E-07 | 2.13E-05 | 0.00010135 |
| xWRvd5Vv35VLuv5Pt0 | 3925 | STMN1 | stathmin 1/oncoprotein 18 | 2.5245 | 2.1914 | 5.86E-08 | 1.52E-07 | 8.77E-06 | 6.78E-05 |
| QdJBAU6BxSNjx.qvnk | 699 | BUB1 | BUB1 budding uninhibited by benzimidazoles 1 homolog (yeast) | 2.4907 | 2.3474 | 2.85E-05 | 4.15E-05 | 0.00030429 | 0.0013758 |
| oq6CuezcH7vXXCvO1w | 55388 | MCM10 | minichromosome maintenance complex component 10 | 2.4888 | 2.7297 | 2.32E-07 | 1.33E-07 | 1.66E-05 | 6.15E-05 |
| lgUgXRN_e9aZUcVCAU | 1163 | CKS1B | CDC28 protein kinase regulatory subunit 1B | 2.479 | 2.2787 | 1.86E-07 | 3.26E-07 | 1.51E-05 | 0.00010017 |
| Ey6v5_K6wp.noV75KQ | 6240 | RRM1 | ribonucleotide reductase M1 | 2.4357 | 2.8658 | 3.37E-06 | 1.30E-06 | 7.97E-05 | 0.00018009 |
| NhXndz1IrriqOQIHEo | 10615 | SPAG5 | sperm associated antigen 5 | 2.4295 | 2.2748 | 2.02E-07 | 3.15E-07 | 1.58E-05 | 9.96E-05 |
| ioJIoIo4AO5S4n4tfo | 10403 | NDC80 | NDC80 homolog, kinetochore complex component (S. cerevisiae) | 2.42 | 2.1917 | 5.79E-06 | 1.13E-05 | 0.00010719 | 0.00063768 |
| ovtEinu7lcR4Uq.sAU | 144455 | E2F7 | E2F transcription factor 7 | 2.4175 | 2.2931 | 9.06E-08 | 1.29E-07 | 1.06E-05 | 6.15E-05 |
| c1ILCUnujVlR0gCmII | 23649 | POLA2 | polymerase (DNA directed), alpha 2 (70kD subunit) | 2.4035 | 2.1421 | 3.62E-06 | 8.05E-06 | 8.25E-05 | 0.0005441 |
| igFXtVLSIwTUHB1IJo | 9156 | EXO1 | exonuclease 1 | 2.3978 | 2.2076 | 1.29E-06 | 2.27E-06 | 4.33E-05 | 0.00024027 |
| ooKJT36B6GH3gB6KGI | 83461 | CDCA3 | cell division cycle associated 3 | 2.3739 | 2.2875 | 1.54E-06 | 1.98E-06 | 4.85E-05 | 0.0002309 |
| lRA1XkJz0Drjoxn6T0 | 259266 | ASPM | asp (abnormal spindle) homolog, microcephaly associated (Drosophila) | 2.3581 | 2.4522 | 2.64E-06 | 2.05E-06 | 6.94E-05 | 0.00023195 |
| up5V906fbnh1MBPd74 | 79019 | CENPM | centromere protein M | 2.3437 | 2.8308 | 9.79E-07 | 3.11E-07 | 3.82E-05 | 9.96E-05 |
| u3o7h3vFf9UQjKKg1U | 79000 | C1orf135 | chromosome 1 open reading frame 135 | 2.3391 | 2.0824 | 1.29E-07 | 3.00E-07 | 1.23E-05 | 9.96E-05 |
| lLkJVIh77K_f.fXXqQ | 150468 | CKAP2L | cytoskeleton associated protein 2-like | 2.3312 | 2.5606 | 5.60E-07 | 3.06E-07 | 2.77E-05 | 9.96E-05 |
| QLR0VHu.euUKd_KlUc | 54478 | FAM64A | family with sequence similarity 64, member A | 2.3108 | 2.1022 | 1.30E-05 | 2.55E-05 | 0.00018291 | 0.00099989 |
| lV.9_MUdNRu9ePtRP0 | 10635 | RAD51AP1 | RAD51 associated protein 1 | 2.3005 | 2.827 | 1.31E-06 | 3.70E-07 | 4.37E-05 | 0.00010851 |
| 9q1S_vuSkou33NLzF0 | 10733 | PLK4 | polo-like kinase 4 (Drosophila) | 2.2514 | 2.3289 | 0.00027968 | 0.00022473 | 0.0015698 | 0.0039704 |
| o7h_frpdPU7uXuXqk4 | 55143 | CDCA8 | cell division cycle associated 8 | 2.2385 | 2.0062 | 6.48E-07 | 1.49E-06 | 2.97E-05 | 0.00019485 |
| TRPIdFYoP8ASXM5UqU | 55635 | DEPDC1 | DEP domain containing 1 | 2.2314 | 2.3358 | 1.51E-05 | 1.11E-05 | 0.00020225 | 0.00063768 |
| 9d5S3L96kr9e7Sz1V0 | 83879 | CDCA7 | cell division cycle associated 7 | 2.2171 | 3.5519 | 9.54E-06 | 6.77E-07 | 0.00014898 | 0.00014371 |
| 0dFB5FLiggh5uj06ok | 22974 | TPX2 | TPX2, microtubule-associated, homolog (Xenopus laevis) | 2.2089 | 2.3195 | 3.42E-06 | 2.43E-06 | 8.02E-05 | 0.00025048 |
| Tb_naZrptCZP1rfNzg | 8364 | HIST1H4C | histone cluster 1, H4c | 2.1971 | 2.0581 | 0.00028664 | 0.00045394 | 0.0015977 | 0.0062104 |
| NH1MoTHk7CULTog3nk | 891 | CCNB1 | cyclin B1 | 2.185 | 2.5001 | 1.20E-07 | 4.81E-08 | 1.19E-05 | 4.41E-05 |
| rBVAPRekS0fRC_3Fl4 | 1434 | CSE1L | CSE1 chromosome segregation 1-like (yeast) | 2.1832 | 2.5333 | 9.11E-06 | 3.39E-06 | 0.0001451 | 0.00032453 |
| f0gC47oTKKQ7uIfqr0 | 54443 | ANLN | anillin, actin binding protein | 2.1674 | 3.1127 | 1.40E-05 | 1.58E-06 | 0.0001923 | 0.00020058 |
| N4TSXn31Xeaf0eeL94 | 7083 | TK1 | thymidine kinase 1, soluble | 2.1649 | 2.3987 | 1.21E-06 | 5.95E-07 | 4.20E-05 | 0.00013976 |
| lokghwIgYonhSjeYYU | 10460 | TACC3 | transforming, acidic coiled-coil containing protein 3 | 2.1434 | 2.4681 | 2.75E-05 | 1.06E-05 | 0.00029722 | 0.00062777 |
| BAZH7.TkomqQsK_JeE | 5985 | RFC5 | replication factor C (activator 1) 5, 36.5kDa | 2.1349 | 2.6586 | 1.86E-05 | 4.43E-06 | 0.00023016 | 0.00037936 |
| 0vlRHut8sjD_rrHgkU | 122769 | PPIL5 | peptidylprolyl isomerase (cyclophilin)-like 5 | 2.0731 | 2.1586 | 7.84E-07 | 5.76E-07 | 3.41E-05 | 0.00013957 |
| flKJDf5foHe5IMQIyY | 91057 | CCDC34 | coiled-coil domain containing 34 | 2.054 | 2.1672 | 8.64E-07 | 5.73E-07 | 3.58E-05 | 0.00013957 |
| 9jpCddAlL7AMrVeXrE | 26271 | FBXO5 | F-box protein 5 | 2.0441 | 2.2969 | 1.98E-05 | 8.47E-06 | 0.00023998 | 0.00056407 |
| 6W70h16.fUp8S6E00k | 81610 | FAM83D | family with sequence similarity 83, member D | 2.0395 | 2.1424 | 8.79E-05 | 6.09E-05 | 0.00066679 | 0.0017704 |
| lo1eNU.13htRdcVTKI | 1017 | CDK2 | cyclin-dependent kinase 2 | 2.0142 | 2.3441 | 4.96E-06 | 1.63E-06 | 9.81E-05 | 0.00020391 |
| ZVChN4dVxd.O3lS7Po | 5427 | POLE2 | polymerase (DNA directed), epsilon 2 (p59 subunit) | 2.0137 | 2.0862 | 1.77E-05 | 1.35E-05 | 0.00022363 | 0.0007037 |
| NkGdd4Dn7f.3vlgMno | 57214 | KIAA1199 | KIAA1199 | -36.648 | -2.1534 | 7.11E-09 | 4.82E-05 | 4.91E-06 | 0.0014933 |
| 6Wwu19TkK1R3K4yEqE | 8613 | PPAP2B | phosphatidic acid phosphatase type 2B | -9.7856 | -2.0752 | 3.46E-07 | 0.00020842 | 2.13E-05 | 0.0038072 |
| 3Vk2XEIHqIeI0WZBWM | 7123 | CLEC3B | C-type lectin domain family 3, member B | -8.6493 | -2.3863 | 3.69E-09 | 6.88E-07 | 4.43E-06 | 0.00014371 |
| Bl4QMLSSfMVEglLlL4 | 6414 | SEPP1 | selenoprotein P, plasma, 1 | -8.3577 | -2.5786 | 6.53E-08 | 6.60E-06 | 9.07E-06 | 0.00047644 |
| ilrh6EzRdx8DW178mE | 8644 | AKR1C3 | aldo-keto reductase family 1, member C3 (3-alpha hydroxysteroid dehydrogenase, type II) | -6.8965 | -2.5623 | 7.46E-09 | 4.67E-07 | 4.91E-06 | 0.0001312 |
| EbqUp7uI76O.rHtTt4 | 23452 | ANGPTL2 | angiopoietin-like 2 | -5.9044 | -2.2538 | 2.24E-08 | 1.98E-06 | 6.64E-06 | 0.0002309 |
| ukpKBLurg_c6brC5Us | 216 | ALDH1A1 | aldehyde dehydrogenase 1 family, member A1 | -5.817 | -2.1355 | 5.05E-08 | 6.25E-06 | 8.16E-06 | 0.0004611 |
| TnoszXhUhcYT4I.klo | 10924 | SMPDL3A | sphingomyelin phosphodiesterase, acid-like 3A | -5.0113 | -2.2387 | 2.71E-07 | 1.40E-05 | 1.80E-05 | 0.00072128 |
| BS4MBTTflHFV14JRJI | 6387 | CXCL12 | chemokine (C-X-C motif) ligand 12 (stromal cell-derived factor 1) | -4.6546 | -2.0039 | 5.06E-06 | 0.00040535 | 9.85E-05 | 0.0057974 |
| Ka9e9I4Tqgjjr0lHrE | 8988 | HSPB3 | heat shock 27kDa protein 3 | -4.3668 | -2.2661 | 1.22E-06 | 3.39E-05 | 4.20E-05 | 0.0011978 |
| T_HqiKToggHdPSqdSg | 5737 | PTGFR | prostaglandin F receptor (FP) | -3.9269 | -2.0149 | 2.48E-07 | 1.12E-05 | 1.69E-05 | 0.00063768 |
| o4VUSopKAElIJRfhH8 | 9249 | DHRS3 | dehydrogenase/reductase (SDR family) member 3 | -3.5679 | -2.3721 | 1.00E-06 | 9.04E-06 | 3.83E-05 | 0.00058339 |
| uHsBTr1JJe9RFVEU0o | 60370 | AVPI1 | arginine vasopressin-induced 1 | -3.5072 | -2.0062 | 2.85E-06 | 7.76E-05 | 7.27E-05 | 0.0020621 |
| HvXXhL_O7U96l3S58k | 10758 | TRAF3IP2 | TRAF3 interacting protein 2 | -3.4683 | -2.0429 | 2.05E-07 | 4.87E-06 | 1.58E-05 | 0.00040115 |
| 6ut.eiK8ytHXql36tU | 5328 | PLAU | plasminogen activator, urokinase | -2.7283 | 2.7901 | 1.80E-05 | 1.59E-05 | 0.00022567 | 0.00076495 |
| QnnX7JUVOIJCIXwKfU | 55540 | IL17RB | interleukin 17 receptor B | -2.2685 | 3.9851 | 1.27E-06 | 6.28E-08 | 4.31E-05 | 4.54E-05 |
| 6gp1T.eO9ZVHuCLuds | 4885 | NPTX2 | neuronal pentraxin II | -2.078 | 4.3682 | 5.94E-05 | 1.14E-06 | 0.00050504 | 0.00017529 |

| CLID | NAME | Egr1 pathway expression | diffuse-proliferation | inflammatory | limited | normal-like |
| --- | --- | --- | --- | --- | --- | --- |
|  |  |  |  |  |  |  |
| WEE1 | WEE1^WEE1 homolog (S. pombe) | down-regulated | increased | decreased | variable | variable |
| TM4SF1 | TM4SF1^transmembrane 4 L six family member 1 | down-regulated | increased | decreased | variable | variable |
| ADAMTS1 | ADAMTS1^ADAM metallopeptidase with thrombospondin type 1 motif, 1 | down-regulated | increased | decreased | variable | variable |
| PHLDA1 | PHLDA1^pleckstrin homology-like domain, family A, member 1 | down-regulated | increased | decreased | variable | variable |
| BAMBI | BAMBI^BMP and activin membrane-bound inhibitor homolog (Xenopus laevis) | down-regulated | increased | decreased | variable | variable |
| MMP1 | MMP1^matrix metallopeptidase 1 (interstitial collagenase) | down-regulated | increased | decreased | variable | variable |
| STC2 | STC2^stanniocalcin 2 | down-regulated | increased | decreased | variable | variable |
| DKK1 | DKK1^dickkopf homolog 1 (Xenopus laevis) | down-regulated | increased | decreased | variable | variable |
| FOXF2 | FOXF2^forkhead box F2 | down-regulated | increased | decreased | variable | variable |
| IL17RB | IL17RB^interleukin 17 receptor B | down-regulated | increased | decreased | variable | variable |
| HIST1H2BK | HIST1H2BK^histone cluster 1, H2bk | down-regulated | increased | decreased | variable | variable |
| FAM20C | FAM20C^family with sequence similarity 20, member C | down-regulated | increased | decreased | variable | variable |
| AKR1B1 | AKR1B1^aldo-keto reductase family 1, member B1 (aldose reductase) | down-regulated | increased | decreased | variable | variable |
| IGFBP2 | IGFBP2^insulin-like growth factor binding protein 2, 36kDa | down-regulated | increased | decreased | variable | variable |
| HERC5 | HERC5^hect domain and RLD 5 | down-regulated | increased | decreased | variable | variable |
| MMP3 | MMP3^matrix metallopeptidase 3 (stromelysin 1, progelatinase) | down-regulated | increased | decreased | variable | variable |
| HIST1H2BD | HIST1H2BD^histone cluster 1, H2bd | down-regulated | increased | decreased | variable | variable |
| STC1 | STC1^stanniocalcin 1 | down-regulated | increased | decreased | variable | variable |
| CCL20 | CCL20^chemokine (C-C motif) ligand 20 | down-regulated | increased | decreased | variable | variable |
| IL8 | IL8^interleukin 8 | down-regulated | increased | decreased | variable | variable |
| NQO1 | NQO1^NAD(P)H dehydrogenase, quinone 1 | down-regulated | increased | decreased | variable | variable |
| TBX2 | TBX2^T-box 2 | down-regulated | increased | decreased | variable | variable |
| AK3 | AK3^adenylate kinase 3 | down-regulated | increased | decreased | variable | variable |
| PTX3 | PTX3^pentraxin-related gene, rapidly induced by IL-1 beta | down-regulated | increased | decreased | variable | variable |
| CXCL1 | CXCL1^chemokine (C-X-C motif) ligand 1 (melanoma growth stimulating activity, alpha) | down-regulated | increased | decreased | variable | variable |
| MAN1A1 | MAN1A1^mannosidase, alpha, class 1A, member 1 | down-regulated | increased | decreased | variable | variable |
| SOD2 | SOD2^superoxide dismutase 2, mitochondrial | down-regulated | increased | decreased | variable | variable |
| PBEF1 | PBEF1^pre-B-cell colony enhancing factor 1 | down-regulated | increased | decreased | variable | variable |
| TNFRSF10B | TNFRSF10B^tumor necrosis factor receptor superfamily, member 10b | down-regulated | increased | decreased | variable | variable |
| SYTL2 | SYTL2^synaptotagmin-like 2 | down-regulated | increased | decreased | variable | variable |
| NPTX2 | NPTX2^neuronal pentraxin II | down-regulated | increased | decreased | variable | variable |
| GAL | GAL^galanin | down-regulated | increased | decreased | variable | variable |
| SC4MOL | SC4MOL^sterol-C4-methyl oxidase-like | down-regulated | increased | decreased | variable | variable |
| INSIG1 | INSIG1^insulin induced gene 1 | down-regulated |  |  |  |  |
| HMGCS1 | HMGCS1^3-hydroxy-3-methylglutaryl-Coenzyme A synthase 1 (soluble) | down-regulated |  |  |  |  |
| MUC1 | MUC1^mucin 1, cell surface associated | down-regulated |  |  |  |  |
| TERF1 | TERF1^telomeric repeat binding factor (NIMA-interacting) 1 | down-regulated |  |  |  |  |
| SC5DL | SC5DL^sterol-C5-desaturase (ERG3 delta-5-desaturase homolog, S. cerevisiae)-like | down-regulated |  |  |  |  |
| KRTAP1-5 | KRTAP1-5^keratin associated protein 1-5 | down-regulated |  |  |  |  |
| KRTAP1-1 | KRTAP1-1^keratin associated protein 1-1 | down-regulated |  |  |  |  |
| S100A3 | S100A3^S100 calcium binding protein A3 | down-regulated |  |  |  |  |
| VEGFC | VEGFC^vascular endothelial growth factor C | down-regulated |  |  |  |  |
| SOCS2 | SOCS2^suppressor of cytokine signaling 2 | down-regulated |  |  |  |  |
| MEIS2 | MEIS2^Meis homeobox 2 | down-regulated |  |  |  |  |
| OSAP | OSAP^ovary-specific acidic protein | down-regulated |  |  |  |  |
| EPSTI1 | EPSTI1^epithelial stromal interaction 1 (breast) | down-regulated |  |  |  |  |
| UGCG | UGCG^UDP-glucose ceramide glucosyltransferase | down-regulated |  |  |  |  |
| PLEKHA4 | PLEKHA4^pleckstrin homology domain containing, family A (phosphoinositide binding specific) member 4 | down-regulated |  |  |  |  |
| FLJ11286 | FLJ11286^hypothetical protein FLJ11286 | down-regulated |  |  |  |  |
| TNFRSF14 | TNFRSF14^tumor necrosis factor receptor superfamily, member 14 (herpesvirus entry mediator) | down-regulated |  |  |  |  |
| ISGF3G | ISGF3G^interferon-stimulated transcription factor 3, gamma 48kDa | down-regulated |  |  |  |  |
| UBE1L | UBE1L^ubiquitin-activating enzyme E1-like | down-regulated |  |  |  |  |
| RAB3IL1 | RAB3IL1^RAB3A interacting protein (rabin3)-like 1 | down-regulated |  |  |  |  |
| SEMA5A | SEMA5A^sema domain, seven thrombospondin repeats (type 1 and type 1-like), transmembrane domain (TM) and short cytoplasmic domain, (semaphorin) 5A | down-regulated |  |  |  |  |
| BMP2K | BMP2K^BMP2 inducible kinase | down-regulated |  |  |  |  |
| HSD17B2 | HSD17B2^hydroxysteroid (17-beta) dehydrogenase 2 | down-regulated |  |  |  |  |
| PSME1 | PSME1^proteasome (prosome, macropain) activator subunit 1 (PA28 alpha) | down-regulated |  |  |  |  |
| RNASET2 | RNASET2^ribonuclease T2 | down-regulated |  |  |  |  |
| IFIT3 | IFIT3^interferon-induced protein with tetratricopeptide repeats 3 | down-regulated |  |  |  |  |
| OAS2 | OAS2^2'-5'-oligoadenylate synthetase 2, 69/71kDa | down-regulated |  |  |  |  |
| CD68 | CD68^CD68 molecule | down-regulated |  |  |  |  |
| KITLG | KITLG^KIT ligand | down-regulated |  |  |  |  |
| STAT2 | STAT2^signal transducer and activator of transcription 2, 113kDa | down-regulated |  |  |  |  |
| CEBPD | CEBPD^CCAAT/enhancer binding protein (C/EBP), delta | down-regulated |  |  |  |  |
| UBE2L6 | UBE2L6^ubiquitin-conjugating enzyme E2L 6 | down-regulated |  |  |  |  |
| PRIC285 | PRIC285^peroxisomal proliferator-activated receptor A interacting complex 285 | down-regulated |  |  |  |  |
| PARP14 | PARP14^poly (ADP-ribose) polymerase family, member 14 | down-regulated |  |  |  |  |
| CITED2 | CITED2^Cbp/p300-interacting transactivator, with Glu/Asp-rich carboxy-terminal domain, 2 | down-regulated |  |  |  |  |
| RABGAP1 | RABGAP1^RAB GTPase activating protein 1 | down-regulated |  |  |  |  |
| SMAD6 | SMAD6^SMAD family member 6 | down-regulated |  |  |  |  |
| PRDM8 | PRDM8^PR domain containing 8 | down-regulated |  |  |  |  |
| LY6E | LY6E^lymphocyte antigen 6 complex, locus E | down-regulated |  |  |  |  |
| MYLIP | MYLIP^myosin regulatory light chain interacting protein | down-regulated |  |  |  |  |
| SIPA1L2 | SIPA1L2^signal-induced proliferation-associated 1 like 2 | down-regulated |  |  |  |  |
| CYP1B1 | CYP1B1^cytochrome P450, family 1, subfamily B, polypeptide 1 | down-regulated |  |  |  |  |
| SERPINB1 | SERPINB1^serpin peptidase inhibitor, clade B (ovalbumin), member 1 | down-regulated |  |  |  |  |
| TNFRSF11B | TNFRSF11B^tumor necrosis factor receptor superfamily, member 11b (osteoprotegerin) | down-regulated |  |  |  |  |
| KIAA1199 | KIAA1199^KIAA1199 | down-regulated |  |  |  |  |
| FBN2 | FBN2^fibrillin 2 (congenital contractural arachnodactyly) | down-regulated |  |  |  |  |
| DBC1 | DBC1^deleted in bladder cancer 1 | down-regulated |  |  |  |  |
| SMAD3 | SMAD3^SMAD family member 3 | down-regulated |  |  |  |  |
| FST | FST^follistatin | down-regulated |  |  |  |  |
| RHOBTB3 | RHOBTB3^Rho-related BTB domain containing 3 | down-regulated |  |  |  |  |
| IFI35 | IFI35^interferon-induced protein 35 | down-regulated |  |  |  |  |
| IRF1 | IRF1^interferon regulatory factor 1 | down-regulated |  |  |  |  |
| PSEN2 | PSEN2^presenilin 2 (Alzheimer disease 4) | down-regulated |  |  |  |  |
| CLDN11 | CLDN11^claudin 11 (oligodendrocyte transmembrane protein) | down-regulated |  |  |  |  |
| BTBD2 | BTBD2^BTB (POZ) domain containing 2 | down-regulated |  |  |  |  |
| PAPPA | PAPPA^pregnancy-associated plasma protein A, pappalysin 1 | down-regulated |  |  |  |  |
| FAM46C | FAM46C^family with sequence similarity 46, member C | down-regulated |  |  |  |  |
| LSS | LSS^lanosterol synthase (2,3-oxidosqualene-lanosterol cyclase) | down-regulated |  |  |  |  |
| RARRES3 | RARRES3^retinoic acid receptor responder (tazarotene induced) 3 | down-regulated |  |  |  |  |
| COLEC12 | COLEC12^collectin sub-family member 12 | down-regulated |  |  |  |  |
| CNTNAP1 | CNTNAP1^contactin associated protein 1 | down-regulated |  |  |  |  |
| ZNF395 | ZNF395^zinc finger protein 395 | down-regulated |  |  |  |  |
| OLFML1 | OLFML1^olfactomedin-like 1 | down-regulated |  |  |  |  |
| ALDH3B1 | ALDH3B1^aldehyde dehydrogenase 3 family, member B1 | down-regulated |  |  |  |  |
| IGF2 | IGF2^insulin-like growth factor 2 (somatomedin A) | down-regulated |  |  |  |  |
| FTH1 | FTH1^ferritin, heavy polypeptide 1 | down-regulated |  |  |  |  |
| SLC39A8 | SLC39A8^solute carrier family 39 (zinc transporter), member 8 | down-regulated |  |  |  |  |
| FGF13 | FGF13^fibroblast growth factor 13 | down-regulated |  |  |  |  |
| EHBP1 | EHBP1^EH domain binding protein 1 | down-regulated |  |  |  |  |
| GPR15 | GPR15^G protein-coupled receptor 15 | down-regulated |  |  |  |  |
| RPS29 | RPS29^ribosomal protein S29 | down-regulated |  |  |  |  |
| LGALS3 | LGALS3^lectin, galactoside-binding, soluble, 3 | down-regulated |  |  |  |  |
| JAZF1 | JAZF1^JAZF zinc finger 1 | down-regulated |  |  |  |  |
| CDC42EP2 | CDC42EP2^CDC42 effector protein (Rho GTPase binding) 2 | down-regulated |  |  |  |  |
| PTHR1 | PTHR1^parathyroid hormone receptor 1 | down-regulated |  |  |  |  |
| PODN | PODN^podocan | down-regulated |  |  |  |  |
| SELENBP1 | SELENBP1^selenium binding protein 1 | down-regulated |  |  |  |  |
| CRYAB | CRYAB^crystallin, alpha B | down-regulated |  |  |  |  |
| HAPLN3 | HAPLN3^hyaluronan and proteoglycan link protein 3 | down-regulated |  |  |  |  |
| ARSD | ARSD^arylsulfatase D | down-regulated |  |  |  |  |
| TNFAIP3 | TNFAIP3^tumor necrosis factor, alpha-induced protein 3 | down-regulated |  |  |  |  |
| CPA4 | CPA4^carboxypeptidase A4 | down-regulated |  |  |  |  |
| CXCL6 | CXCL6^chemokine (C-X-C motif) ligand 6 (granulocyte chemotactic protein 2) | down-regulated |  |  |  |  |
| LDLR | LDLR^low density lipoprotein receptor (familial hypercholesterolemia) | down-regulated |  |  |  |  |
| FSTL5 | FSTL5^follistatin-like 5 | down-regulated |  |  |  |  |
| TGFBR2 | TGFBR2^transforming growth factor, beta receptor II (70/80kDa) | down-regulated |  |  |  |  |
| IL6ST | IL6ST^interleukin 6 signal transducer (gp130, oncostatin M receptor) | down-regulated |  |  |  |  |
| GPNMB | GPNMB^glycoprotein (transmembrane) nmb | down-regulated |  |  |  |  |
| GLRX | GLRX^glutaredoxin (thioltransferase) | down-regulated | decreased | increased | increased | increased |
| PPARG | PPARG^peroxisome proliferator-activated receptor gamma | down-regulated | decreased | increased | increased | increased |
| CAV1 | CAV1^caveolin 1, caveolae protein, 22kDa | down-regulated | decreased | increased | increased | increased |
| AKR1C3 | AKR1C3^aldo-keto reductase family 1, member C3 (3-alpha hydroxysteroid dehydrogenase, type II) | down-regulated | decreased | increased | increased | increased |
| ADM | ADM^adrenomedullin | down-regulated | decreased | increased | increased | increased |
| SFRP1 | SFRP1^secreted frizzled-related protein 1 | down-regulated | decreased | increased | increased | increased |
| FUCA1 | FUCA1^fucosidase, alpha-L- 1, tissue | down-regulated | decreased | increased | increased | increased |
| NDN | NDN^necdin homolog (mouse) | down-regulated | decreased | increased | increased | increased |
| DIO2 | DIO2^deiodinase, iodothyronine, type II | down-regulated | decreased | increased | increased | increased |
| TWIST1 | TWIST1^twist homolog 1 (acrocephalosyndactyly 3; Saethre-Chotzen syndrome) (Drosophila) | down-regulated | decreased | increased | increased | increased |
| C18orf17 | C18orf17^chromosome 18 open reading frame 17 | down-regulated | decreased | increased | increased | increased |
| SDPR | SDPR^serum deprivation response (phosphatidylserine binding protein) | down-regulated | decreased | increased | increased | increased |
| PRRX2 | PRRX2^paired related homeobox 2 | down-regulated | decreased | increased | increased | increased |
| VAMP5 | VAMP5^vesicle-associated membrane protein 5 (myobrevin) | down-regulated | decreased | increased | increased | increased |
| PYCARD | PYCARD^PYD and CARD domain containing | down-regulated | decreased | increased | increased | increased |
| IGF2R | IGF2R^insulin-like growth factor 2 receptor | down-regulated | decreased | increased | increased | increased |
| KCNJ2 | KCNJ2^potassium inwardly-rectifying channel, subfamily J, member 2 | down-regulated | decreased | increased | increased | increased |
| IFIT2 | IFIT2^interferon-induced protein with tetratricopeptide repeats 2 | down-regulated | decreased | increased | increased | increased |
| IFIT1 | IFIT1^interferon-induced protein with tetratricopeptide repeats 1 | down-regulated | decreased | increased | increased | increased |
| MX1 | MX1^myxovirus (influenza virus) resistance 1, interferon-inducible protein p78 (mouse) | down-regulated | decreased | increased | increased | increased |
| PARP9 | PARP9^poly (ADP-ribose) polymerase family, member 9 | down-regulated | decreased | increased | increased | increased |
| IFIH1 | IFIH1^interferon induced with helicase C domain 1 | down-regulated | decreased | increased | increased | increased |
| STAT1 | STAT1^signal transducer and activator of transcription 1, 91kDa | down-regulated | decreased | increased | increased | increased |
| SP110 | SP110^SP110 nuclear body protein | down-regulated | decreased | increased | increased | increased |
| PMP22 | PMP22^peripheral myelin protein 22 | down-regulated | decreased | increased | increased | increased |
| SERPINB2 | SERPINB2^serpin peptidase inhibitor, clade B (ovalbumin), member 2 | down-regulated | decreased | increased | increased | increased |
| MAP3K5 | MAP3K5^mitogen-activated protein kinase kinase kinase 5 | down-regulated | decreased | increased | increased | increased |
| PPAP2B | PPAP2B^phosphatidic acid phosphatase type 2B | down-regulated | decreased | increased | increased | increased |
| EDG2 | EDG2^endothelial differentiation, lysophosphatidic acid G-protein-coupled receptor, 2 | down-regulated | decreased | increased | increased | increased |
| PDGFRA | PDGFRA^platelet-derived growth factor receptor, alpha polypeptide | down-regulated | decreased | increased | increased | increased |
| ARMCX1 | ARMCX1^armadillo repeat containing, X-linked 1 | down-regulated | decreased | increased | increased | increased |
| LOC153222 | LOC153222^adult retina protein | down-regulated | decreased | increased | increased | increased |
| SERPINB7 | SERPINB7^serpin peptidase inhibitor, clade B (ovalbumin), member 7 | down-regulated | decreased | increased | increased | increased |
| CYBRD1 | CYBRD1^cytochrome b reductase 1 | down-regulated | decreased | increased | increased | increased |
| DCN | DCN^decorin | down-regulated | decreased | increased | increased | increased |
| PLSCR4 | PLSCR4^phospholipid scramblase 4 | down-regulated | decreased | increased | increased | increased |
| ZNF521 | ZNF521^zinc finger protein 521 | down-regulated | decreased | increased | increased | increased |
| PTGFR | PTGFR^prostaglandin F receptor (FP) | down-regulated | decreased | increased | increased | increased |
| GPM6B | GPM6B^glycoprotein M6B | down-regulated | decreased | increased | increased | increased |
| KIT | KIT^v-kit Hardy-Zuckerman 4 feline sarcoma viral oncogene homolog | down-regulated | decreased | increased | increased | increased |
| RECK | RECK^reversion-inducing-cysteine-rich protein with kazal motifs | down-regulated | decreased | increased | increased | increased |
| DPP4 | DPP4^dipeptidyl-peptidase 4 (CD26, adenosine deaminase complexing protein 2) | down-regulated | decreased | increased | increased | increased |
| C9orf103 | C9orf103^chromosome 9 open reading frame 103 | down-regulated | decreased | increased | increased | increased |
| SVIL | SVIL^supervillin | down-regulated | decreased | increased | increased | increased |
| UST | UST^uronyl-2-sulfotransferase | down-regulated | decreased | increased | increased | increased |
| HSPB3 | HSPB3^heat shock 27kDa protein 3 | down-regulated | decreased | increased | increased | increased |
| SQRDL | SQRDL^sulfide quinone reductase-like (yeast) | down-regulated | decreased | increased | increased | increased |
| DPYSL2 | DPYSL2^dihydropyrimidinase-like 2 | down-regulated | decreased | increased | increased | increased |
| MGC7036 | MGC7036^hypothetical protein MGC7036 | down-regulated | decreased | increased | increased | increased |
| PDE5A | PDE5A^phosphodiesterase 5A, cGMP-specific | down-regulated | decreased | increased | increased | increased |
| ALDH1A1 | ALDH1A1^aldehyde dehydrogenase 1 family, member A1 | down-regulated | decreased | increased | increased | increased |
| COBLL1 | COBLL1^COBL-like 1 | down-regulated | decreased | increased | increased | increased |
| EPAS1 | EPAS1^endothelial PAS domain protein 1 | down-regulated | decreased | increased | increased | increased |
| ENPP2 | ENPP2^ectonucleotide pyrophosphatase/phosphodiesterase 2 (autotaxin) | down-regulated | decreased | increased | increased | increased |
| LAMA4 | LAMA4^laminin, alpha 4 | down-regulated | decreased | increased | increased | increased |
| NOV | NOV^nephroblastoma overexpressed gene | down-regulated | decreased | increased | increased | increased |
| STOM | STOM^stomatin | down-regulated | decreased | increased | increased | increased |
| FHL1 | FHL1^four and a half LIM domains 1 | down-regulated | decreased | increased | increased | increased |
| PCDH18 | PCDH18^protocadherin 18 | down-regulated | decreased | increased | increased | increased |
| PTGIS | PTGIS^prostaglandin I2 (prostacyclin) synthase | down-regulated | decreased | increased | increased | increased |
| DPYD | DPYD^dihydropyrimidine dehydrogenase | down-regulated | decreased | increased | increased | increased |
| KIAA0999 | KIAA0999^KIAA0999 protein | down-regulated | decreased | increased | increased | increased |
| FYCO1 | FYCO1^FYVE and coiled-coil domain containing 1 | down-regulated | decreased | increased | increased | increased |
| PAM | PAM^peptidylglycine alpha-amidating monooxygenase | down-regulated | decreased | increased | increased | increased |
| S100A10 | S100A10^S100 calcium binding protein A10 | down-regulated | decreased | increased | increased | increased |
| S100A4 | S100A4^S100 calcium binding protein A4 | down-regulated | decreased | increased | increased | increased |
| CAT | CAT^catalase | down-regulated | decreased | increased | increased | increased |
| PHYH | PHYH^phytanoyl-CoA 2-hydroxylase | down-regulated | decreased | increased | increased | increased |
| MAOA | MAOA^monoamine oxidase A | down-regulated | decreased | increased | increased | increased |
| RDH10 | RDH10^retinol dehydrogenase 10 (all-trans) | down-regulated | decreased | increased | increased | increased |
| ANGPT1 | ANGPT1^angiopoietin 1 | down-regulated | decreased | increased | increased | increased |
| HIBADH | HIBADH^3-hydroxyisobutyrate dehydrogenase | down-regulated | decreased | increased | increased | increased |
| ARHGEF3 | ARHGEF3^Rho guanine nucleotide exchange factor (GEF) 3 | down-regulated | decreased | increased | increased | increased |
| DAAM2 | DAAM2^dishevelled associated activator of morphogenesis 2 | down-regulated | decreased | increased | increased | increased |
| MME | MME^membrane metallo-endopeptidase | down-regulated | decreased | increased | increased | increased |
| CTSO | CTSO^cathepsin O | down-regulated | decreased | increased | increased | increased |
| IL10RB | IL10RB^interleukin 10 receptor, beta | down-regulated | decreased | increased | increased | increased |
| IL1R1 | IL1R1^interleukin 1 receptor, type I | down-regulated | decreased | increased | increased | increased |
| AOX1 | AOX1^aldehyde oxidase 1 | down-regulated | decreased | increased | increased | increased |
| C6orf72 | C6orf72^chromosome 6 open reading frame 72 | down-regulated | decreased | increased | increased | increased |
| CH25H | CH25H^cholesterol 25-hydroxylase | down-regulated | decreased | increased | increased | increased |
| COL21A1 | COL21A1^collagen, type XXI, alpha 1 | down-regulated | decreased | increased | increased | increased |
| FLJ20160 | FLJ20160^FLJ20160 protein | down-regulated | decreased | increased | increased | increased |
| SLC15A3 | SLC15A3^solute carrier family 15, member 3 | down-regulated | decreased | increased | increased | increased |
| CTSK | CTSK^cathepsin K | down-regulated | decreased | increased | increased | increased |
| TRIM22 | TRIM22^tripartite motif-containing 22 | down-regulated | decreased | increased | increased | increased |
| LAMA2 | LAMA2^laminin, alpha 2 (merosin, congenital muscular dystrophy) | down-regulated | decreased | increased | increased | increased |
| ADAMTS5 | ADAMTS5^ADAM metallopeptidase with thrombospondin type 1 motif, 5 (aggrecanase-2) | down-regulated | decreased | increased | increased | increased |
| C1R | C1R^complement component 1, r subcomponent | down-regulated | decreased | increased | increased | increased |
| CCL8 | CCL8^chemokine (C-C motif) ligand 8 | down-regulated | decreased | increased | increased | increased |
| CPXM2 | CPXM2^carboxypeptidase X (M14 family), member 2 | down-regulated | decreased | increased | increased | increased |
| HCST | HCST^hematopoietic cell signal transducer | down-regulated | decreased | increased | increased | increased |
| C1RL | C1RL^complement component 1, r subcomponent-like | down-regulated | decreased | increased | increased | increased |
| EPB41L3 | EPB41L3^erythrocyte membrane protein band 4.1-like 3 | down-regulated | decreased | increased | increased | increased |
| BHLHB5 | BHLHB5^basic helix-loop-helix domain containing, class B, 5 | down-regulated | decreased | increased | increased | increased |
| CTSB | CTSB^cathepsin B | down-regulated | decreased | increased | increased | increased |
| CCL2 | CCL2^chemokine (C-C motif) ligand 2 | down-regulated | decreased | increased | increased | increased |
| CTSC | CTSC^cathepsin C | down-regulated | decreased | increased | increased | increased |
| SRPX | SRPX^sushi-repeat-containing protein, X-linked | down-regulated | decreased | increased | increased | increased |
| SEPP1 | SEPP1^selenoprotein P, plasma, 1 | down-regulated | decreased | increased | increased | increased |
| BOC | BOC^Boc homolog (mouse) | down-regulated | decreased | increased | increased | variable |
| DHRS3 | DHRS3^dehydrogenase/reductase (SDR family) member 3 | down-regulated | decreased | increased | increased | variable |
| RGL1 | RGL1^ral guanine nucleotide dissociation stimulator-like 1 | down-regulated | decreased | increased | increased | variable |
| CDC42EP4 | CDC42EP4^CDC42 effector protein (Rho GTPase binding) 4 | down-regulated | decreased | increased | increased | variable |
| OSR2 | OSR2^odd-skipped related 2 (Drosophila) | down-regulated | decreased | increased | increased | variable |
| MRGPRF | MRGPRF^MAS-related GPR, member F | down-regulated | decreased | increased | increased | variable |
| SLC24A6 | SLC24A6^solute carrier family 24 (sodium/potassium/calcium exchanger), member 6 | down-regulated | decreased | increased | increased | variable |
| IGFBP5 | IGFBP5^insulin-like growth factor binding protein 5 | down-regulated | decreased | increased | increased | variable |
| MASP1 | MASP1^mannan-binding lectin serine peptidase 1 (C4/C2 activating component of Ra-reactive factor) | down-regulated | decreased | increased | increased | variable |
| DDEFL1 | DDEFL1^development and differentiation enhancing factor-like 1 | down-regulated | decreased | increased | increased | variable |
| APCDD1 | APCDD1^adenomatosis polyposis coli down-regulated 1 | down-regulated | decreased | increased | increased | variable |
| C14orf159 | C14orf159^chromosome 14 open reading frame 159 | down-regulated | decreased | increased | increased | variable |
| ANGPTL2 | ANGPTL2^angiopoietin-like 2 | down-regulated | decreased | increased | increased | variable |
| FCGRT | FCGRT^Fc fragment of IgG, receptor, transporter, alpha | down-regulated | decreased | increased | increased | variable |
| RPS6KA2 | RPS6KA2^ribosomal protein S6 kinase, 90kDa, polypeptide 2 | down-regulated | decreased | increased | increased | variable |
| LDB2 | LDB2^LIM domain binding 2 | down-regulated | decreased | increased | increased | variable |
| ALPL | ALPL^alkaline phosphatase, liver/bone/kidney | down-regulated | decreased | increased | increased | variable |
| PDGFD | PDGFD^platelet derived growth factor D | down-regulated | decreased | increased | increased | variable |
| CLDN23 | CLDN23^claudin 23 | down-regulated | decreased | increased | increased | variable |
| GAS1 | GAS1^growth arrest-specific 1 | down-regulated | decreased | increased | increased | variable |
| CCRL1 | CCRL1^chemokine (C-C motif) receptor-like 1 | down-regulated | decreased | increased | increased | variable |
| SETBP1 | SETBP1^SET binding protein 1 | down-regulated | decreased | increased | increased | variable |
| LRIG3 | LRIG3^leucine-rich repeats and immunoglobulin-like domains 3 | down-regulated | decreased | increased | increased | variable |
| TGFBR3 | TGFBR3^transforming growth factor, beta receptor III | down-regulated | decreased | increased | increased | variable |
| SERPING1 | SERPING1^serpin peptidase inhibitor, clade G (C1 inhibitor), member 1, (angioedema, hereditary) | down-regulated | decreased | increased | increased | variable |
| APOD | APOD^apolipoprotein D | down-regulated | decreased | increased | increased | variable |
| ADH1A | ADH1A^alcohol dehydrogenase 1A (class I), alpha polypeptide | down-regulated | decreased | increased | increased | variable |
| SLIT2 | SLIT2^slit homolog 2 (Drosophila) | down-regulated | decreased | increased | increased | variable |
| LHFP | LHFP^lipoma HMGIC fusion partner | down-regulated | decreased | increased | increased | variable |
| PROS1 | PROS1^protein S (alpha) | down-regulated | decreased | increased | increased | variable |
| SLC9A9 | SLC9A9^solute carrier family 9 (sodium/hydrogen exchanger), member 9 | down-regulated | decreased | increased | increased | variable |
| TCEA3 | TCEA3^transcription elongation factor A (SII), 3 | down-regulated | decreased | increased | increased | variable |
| MATN2 | MATN2^matrilin 2 | down-regulated | decreased | increased | increased | variable |
| SMARCA2 | SMARCA2^SWI/SNF related, matrix associated, actin dependent regulator of chromatin, subfamily a, member 2 | down-regulated | decreased | increased | increased | variable |
| SLIT3 | SLIT3^slit homolog 3 (Drosophila) | down-regulated | decreased | increased | increased | variable |
| CXCL12 | CXCL12^chemokine (C-X-C motif) ligand 12 (stromal cell-derived factor 1) | down-regulated | decreased | increased | increased | variable |
| CLIC6 | CLIC6^chloride intracellular channel 6 | down-regulated | decreased | increased | increased | variable |
| LPPR4 | LPPR4^plasticity related gene 1 | down-regulated | decreased | increased | increased | variable |
| GBP2 | GBP2^guanylate binding protein 2, interferon-inducible | down-regulated | decreased | increased | increased | variable |
| ADCY4 | ADCY4^adenylate cyclase 4 | down-regulated | decreased | increased | increased | variable |
| C10orf116 | C10orf116^chromosome 10 open reading frame 116 | down-regulated | decreased | increased | increased | variable |
| EFEMP1 | EFEMP1^EGF-containing fibulin-like extracellular matrix protein 1 | down-regulated | decreased | increased | increased | variable |
| FMOD | FMOD^fibromodulin | down-regulated | decreased | increased | increased | variable |
| GLI3 | GLI3^GLI-Kruppel family member GLI3 (Greig cephalopolysyndactyly syndrome) | down-regulated | decreased | increased | increased | variable |
| FBLN2 | FBLN2^fibulin 2 | down-regulated | decreased | increased | increased | variable |
| PDGFRL | PDGFRL^platelet-derived growth factor receptor-like | down-regulated | decreased | increased | increased | variable |
| FBLN1 | FBLN1^fibulin 1 | down-regulated | decreased | increased | increased | variable |
| C1S | C1S^complement component 1, s subcomponent | down-regulated | decreased | increased | increased | variable |
| SERPINF1 | SERPINF1^serpin peptidase inhibitor, clade F (alpha-2 antiplasmin, pigment epithelium derived factor), member 1 | down-regulated | decreased | increased | increased | variable |
| SFRP2 | SFRP2^secreted frizzled-related protein 2 | down-regulated | decreased | increased | increased | variable |
| PTGS1 | PTGS1^prostaglandin-endoperoxide synthase 1 (prostaglandin G/H synthase and cyclooxygenase) | down-regulated | decreased | increased | increased | variable |
| NOPE | NOPE^neighbor of Punc E11 | down-regulated | decreased | increased | increased | increased |
| HSPA2 | HSPA2^heat shock 70kDa protein 2 | down-regulated | decreased | increased | increased | increased |
| GPR1 | GPR1^G protein-coupled receptor 1 | down-regulated | decreased | increased | increased | increased |
| GADD45A | GADD45A^growth arrest and DNA-damage-inducible, alpha | down-regulated | decreased | increased | increased | increased |
| NFIL3 | NFIL3^nuclear factor, interleukin 3 regulated | down-regulated | decreased | increased | increased | increased |
| PLSCR1 | PLSCR1^phospholipid scramblase 1 | down-regulated | decreased | increased | increased | increased |
| LIPA | LIPA^lipase A, lysosomal acid, cholesterol esterase (Wolman disease) | down-regulated | decreased | increased | increased | increased |
| ARL6IP5 | ARL6IP5^ADP-ribosylation-like factor 6 interacting protein 5 | down-regulated | decreased | increased | increased | increased |
| SDCBP | SDCBP^syndecan binding protein (syntenin) | down-regulated | decreased | increased | increased | increased |
| SGCE | SGCE^sarcoglycan, epsilon | down-regulated | decreased | increased | increased | increased |
| CASP1 | CASP1^caspase 1, apoptosis-related cysteine peptidase (interleukin 1, beta, convertase) | down-regulated | decreased | increased | increased | increased |
| IFNGR1 | IFNGR1^interferon gamma receptor 1 | down-regulated | decreased | increased | increased | increased |
| KLF9 | KLF9^Kruppel-like factor 9 | down-regulated | decreased | increased | increased | increased |
| SMPDL3A | SMPDL3A^sphingomyelin phosphodiesterase, acid-like 3A | down-regulated | decreased | increased | increased | increased |
| WTAP | WTAP^Wilms tumor 1 associated protein | down-regulated | decreased | increased | increased | increased |
| RAB9A | RAB9A^RAB9A, member RAS oncogene family | down-regulated | decreased | increased | increased | increased |
| C2orf33 | C2orf33^chromosome 2 open reading frame 33 | down-regulated | decreased | increased | increased | increased |
| ANAPC13 | ANAPC13^anaphase promoting complex subunit 13 | down-regulated | decreased | increased | increased | increased |
| RAB11FIP2 | RAB11FIP2^RAB11 family interacting protein 2 (class I) | down-regulated | decreased | increased | increased | increased |
| REV3L | REV3L^REV3-like, catalytic subunit of DNA polymerase zeta (yeast) | down-regulated | decreased | increased | increased | increased |
| ZAK | ZAK^sterile alpha motif and leucine zipper containing kinase AZK | down-regulated | decreased | increased | increased | increased |
| C8orf4 | C8orf4^chromosome 8 open reading frame 4 | down-regulated | decreased | increased | increased | increased |
| ALDH1A3 | ALDH1A3^aldehyde dehydrogenase 1 family, member A3 | down-regulated | decreased | increased | increased | increased |
| ZFP36 | ZFP36^zinc finger protein 36, C3H type, homolog (mouse) | down-regulated | decreased | increased | increased | increased |
| EGR1 | EGR1^early growth response 1 | down-regulated | decreased | increased | increased | increased |
| NFKBIA | NFKBIA^nuclear factor of kappa light polypeptide gene enhancer in B-cells inhibitor, alpha | down-regulated | decreased | increased | increased | increased |
| TNFRSF19 | TNFRSF19^tumor necrosis factor receptor superfamily, member 19 | down-regulated | decreased | increased | increased | increased |
| COL4A5 | COL4A5^collagen, type IV, alpha 5 (Alport syndrome) | down-regulated | decreased | increased | increased | increased |
| KLF4 | KLF4^Kruppel-like factor 4 (gut) | down-regulated | decreased | increased | increased | increased |
| ALDH3A2 | ALDH3A2^aldehyde dehydrogenase 3 family, member A2 | down-regulated | decreased | increased | increased | increased |
| CRIP1 | CRIP1^cysteine-rich protein 1 (intestinal) | down-regulated | decreased | increased | increased | increased |
| PLEKHA5 | PLEKHA5^pleckstrin homology domain containing, family A member 5 | down-regulated | decreased | increased | increased | increased |
| ADORA2B | ADORA2B^adenosine A2b receptor | down-regulated | decreased | increased | increased | increased |
| MAP7 | MAP7^microtubule-associated protein 7 | down-regulated | decreased | increased | increased | increased |
| RAB38 | RAB38^RAB38, member RAS oncogene family | down-regulated | decreased | increased | increased | increased |
| ADFP | ADFP^adipose differentiation-related protein | down-regulated | decreased | increased | increased | increased |
| PCOLCE2 | PCOLCE2^procollagen C-endopeptidase enhancer 2 | down-regulated | decreased | increased | increased | increased |
| HSD11B1 | HSD11B1^hydroxysteroid (11-beta) dehydrogenase 1 | down-regulated | decreased | increased | increased | increased |
| DHCR24 | DHCR24^24-dehydrocholesterol reductase | down-regulated | decreased | increased | increased | increased |
| C6orf32 | C6orf32^chromosome 6 open reading frame 32 | down-regulated | decreased | increased | increased | increased |
| PTGES | PTGES^prostaglandin E synthase | down-regulated | decreased | increased | increased | increased |
| CXCL2 | CXCL2^chemokine (C-X-C motif) ligand 2 | down-regulated | decreased | increased | increased | increased |
| ISG20 | ISG20^interferon stimulated exonuclease gene 20kDa | down-regulated | decreased | increased | increased | increased |
| IFI44 | IFI44^interferon-induced protein 44 | down-regulated | decreased | increased | increased | increased |
| C1QTNF6 | C1QTNF6^C1q and tumor necrosis factor related protein 6 | down-regulated | decreased | increased | increased | increased |
| PLAU | PLAU^plasminogen activator, urokinase | down-regulated | decreased | increased | increased | increased |
| IFITM1 | IFITM1^interferon induced transmembrane protein 1 (9-27) | down-regulated | decreased | increased | increased | increased |
| IFITM3 | IFITM3^interferon induced transmembrane protein 3 (1-8U) | down-regulated | decreased | increased | increased | increased |
| PSMB10 | PSMB10^proteasome (prosome, macropain) subunit, beta type, 10 | down-regulated | decreased | increased | increased | increased |
| LYN | LYN^v-yes-1 Yamaguchi sarcoma viral related oncogene homolog | down-regulated | decreased | increased | increased | increased |
| GPX1 | GPX1^glutathione peroxidase 1 | down-regulated | decreased | increased | increased | increased |
| PRCP | PRCP^prolylcarboxypeptidase (angiotensinase C) | down-regulated | decreased | increased | increased | increased |
| SULF1 | SULF1^sulfatase 1 | down-regulated | decreased | increased | increased | increased |
| COL8A1 | COL8A1^collagen, type VIII, alpha 1 | down-regulated | decreased | increased | increased | increased |
| LUM | LUM^lumican | down-regulated | decreased | increased | increased | increased |
| SULF2 | SULF2^sulfatase 2 | down-regulated | decreased | increased | increased | increased |
| TFPI | TFPI^tissue factor pathway inhibitor (lipoprotein-associated coagulation inhibitor) | down-regulated | decreased | increased | increased | increased |
| LRAP | LRAP^leukocyte-derived arginine aminopeptidase | down-regulated | decreased | increased | increased | increased |
| C3 | C3^complement component 3 | down-regulated | decreased | increased | increased | increased |
| PTGDS | PTGDS^prostaglandin D2 synthase 21kDa (brain) | down-regulated | decreased | increased | increased | increased |
| TRPC6 | TRPC6^transient receptor potential cation channel, subfamily C, member 6 | down-regulated | decreased | increased | increased | increased |
| MBP | MBP^myelin basic protein | down-regulated | decreased | increased | increased | increased |
| FGF9 | FGF9^fibroblast growth factor 9 (glia-activating factor) | down-regulated | decreased | increased | increased | increased |
| CXCL14 | CXCL14^chemokine (C-X-C motif) ligand 14 | down-regulated | decreased | increased | increased | increased |
| CUEDC1 | CUEDC1^CUE domain containing 1 | down-regulated | decreased | increased | increased | increased |
| ANPEP | ANPEP^alanyl (membrane) aminopeptidase (aminopeptidase N, aminopeptidase M, microsomal aminopeptidase, CD13, p150) | down-regulated | decreased | increased | increased | increased |
| HMOX1 | HMOX1^heme oxygenase (decycling) 1 | down-regulated | decreased | increased | increased | increased |
| FAM43A | FAM43A^family with sequence similarity 43, member A | down-regulated | variable | decreased | increased | decreased |
| GSDMDC1 | GSDMDC1^gasdermin domain containing 1 | down-regulated | variable | decreased | increased | decreased |
| CORO1B | CORO1B^coronin, actin binding protein, 1B | down-regulated | variable | decreased | increased | decreased |
| LAMA5 | LAMA5^laminin, alpha 5 | down-regulated | variable | decreased | increased | decreased |
| SGSH | SGSH^N-sulfoglucosamine sulfohydrolase (sulfamidase) | down-regulated | variable | decreased | increased | decreased |
| RHBDF1 | RHBDF1^rhomboid 5 homolog 1 (Drosophila) | down-regulated | variable | decreased | increased | decreased |
| IGFBP4 | IGFBP4^insulin-like growth factor binding protein 4 | down-regulated | variable | decreased | increased | decreased |
| BMP4 | BMP4^bone morphogenetic protein 4 | down-regulated | variable | decreased | increased | decreased |
| HLA-E | HLA-E^major histocompatibility complex, class I, E | down-regulated | variable | decreased | increased | decreased |
| HLA-B | HLA-B^major histocompatibility complex, class I, B | down-regulated | variable | decreased | increased | decreased |
| GLTSCR2 | GLTSCR2^glioma tumor suppressor candidate region gene 2 | down-regulated | variable | decreased | increased | decreased |
| LMNA | LMNA^lamin A/C | down-regulated | variable | decreased | increased | decreased |
| TRIP6 | TRIP6^thyroid hormone receptor interactor 6 | down-regulated | variable | decreased | increased | decreased |
| HSPA1A | HSPA1A^heat shock 70kDa protein 1A | down-regulated | variable | decreased | increased | decreased |
| EPHB6 | EPHB6^EPH receptor B6 | down-regulated | variable | decreased | increased | decreased |
| MXD4 | MXD4^MAX dimerization protein 4 | down-regulated | variable | decreased | increased | decreased |
| TRIM47 | TRIM47^tripartite motif-containing 47 | down-regulated | variable | decreased | increased | decreased |
| PCOLCE | PCOLCE^procollagen C-endopeptidase enhancer | down-regulated | variable | decreased | increased | decreased |
| LTBR | LTBR^lymphotoxin beta receptor (TNFR superfamily, member 3) | down-regulated | variable | decreased | increased | decreased |
| RALGDS | RALGDS^ral guanine nucleotide dissociation stimulator | down-regulated | variable | decreased | increased | decreased |
| CECR1 | CECR1^cat eye syndrome chromosome region, candidate 1 | down-regulated | variable | decreased | increased | decreased |
| C14orf132 | C14orf132^chromosome 14 open reading frame 132 | down-regulated | variable | decreased | increased | decreased |
| SEMA3B | SEMA3B^sema domain, immunoglobulin domain (Ig), short basic domain, secreted, (semaphorin) 3B | down-regulated | variable | decreased | increased | decreased |
| F10 | F10^coagulation factor X | down-regulated | variable | decreased | increased | decreased |
| AVPI1 | AVPI1^arginine vasopressin-induced 1 | down-regulated | decreased | decreased | increased | decreased |
| LTBP4 | LTBP4^latent transforming growth factor beta binding protein 4 | down-regulated | decreased | decreased | increased | decreased |
| GPX4 | GPX4^glutathione peroxidase 4 (phospholipid hydroperoxidase) | down-regulated | decreased | decreased | increased | decreased |
| EGFL6 | EGFL6^EGF-like-domain, multiple 6 | down-regulated | decreased | decreased | increased | decreased |
| CRABP2 | CRABP2^cellular retinoic acid binding protein 2 | down-regulated | decreased | decreased | increased | decreased |
| FNBP1 | FNBP1^formin binding protein 1 | down-regulated | decreased | decreased | increased | decreased |
| CAPN5 | CAPN5^calpain 5 | down-regulated | decreased | decreased | increased | decreased |
| HIST1H4C | HIST1H4C^histone cluster 1, H4c | up-regulated | decreased | increased | variable | variable |
| MSC | MSC^musculin (activated B-cell factor-1) | up-regulated | decreased | increased | variable | variable |
| AQP1 | AQP1^aquaporin 1 (Colton blood group) | up-regulated | decreased | increased | variable | variable |
| RHOB | RHOB^ras homolog gene family, member B | up-regulated | decreased | increased | variable | variable |
| FLJ14213 | FLJ14213^hypothetical protein FLJ14213 | up-regulated | decreased | increased | variable | variable |
| EGR2 | EGR2^early growth response 2 (Krox-20 homolog, Drosophila) | up-regulated | decreased | increased | variable | variable |
| IL1RN | IL1RN^interleukin 1 receptor antagonist | up-regulated | decreased | increased | variable | variable |
| PRICKLE2 | PRICKLE2^prickle homolog 2 (Drosophila) | up-regulated | decreased | increased | variable | variable |
| COL22A1 | COL22A1^collagen, type XXII, alpha 1 | up-regulated | decreased | increased | variable | variable |
| BTBD11 | BTBD11^BTB (POZ) domain containing 11 | up-regulated | decreased | increased | variable | variable |
| SGCD | SGCD^sarcoglycan, delta (35kDa dystrophin-associated glycoprotein) | up-regulated | decreased | increased | variable | variable |
| LRRN3 | LRRN3^leucine rich repeat neuronal 3 | up-regulated | decreased | increased | variable | variable |
| TUFT1 | TUFT1^tuftelin 1 | up-regulated | decreased | increased | variable | variable |
| PLEK2 | PLEK2^pleckstrin 2 | up-regulated | decreased | increased | variable | variable |
| PPP1R14C | PPP1R14C^protein phosphatase 1, regulatory (inhibitor) subunit 14C | up-regulated | decreased | increased | variable | variable |
| PRDM1 | PRDM1^PR domain containing 1, with ZNF domain | up-regulated | decreased | increased | variable | variable |
| PPP1R13L | PPP1R13L^protein phosphatase 1, regulatory (inhibitor) subunit 13 like | up-regulated | decreased | increased | variable | variable |
| OSBPL6 | OSBPL6^oxysterol binding protein-like 6 | up-regulated | decreased | increased | variable | variable |
| EHD4 | EHD4^EH-domain containing 4 | up-regulated | decreased | increased | variable | variable |
| SEMA7A | SEMA7A^semaphorin 7A, GPI membrane anchor (John Milton Hagen blood group) | up-regulated | decreased | increased | variable | variable |
| TTL | TTL^tubulin tyrosine ligase | up-regulated | decreased | increased | variable | variable |
| S100A16 | S100A16^S100 calcium binding protein A16 | up-regulated | decreased | increased | variable | variable |
| UBL3 | UBL3^ubiquitin-like 3 | up-regulated | decreased | increased | variable | variable |
| ALS2CR4 | ALS2CR4^amyotrophic lateral sclerosis 2 (juvenile) chromosome region, candidate 4 | up-regulated | decreased | increased | variable | variable |
| LGR4 | LGR4^leucine-rich repeat-containing G protein-coupled receptor 4 | up-regulated | decreased | increased | variable | variable |
| LIF | LIF^leukemia inhibitory factor (cholinergic differentiation factor) | up-regulated | decreased | increased | variable | variable |
| GFPT2 | GFPT2^glutamine-fructose-6-phosphate transaminase 2 | up-regulated | decreased | increased | variable | variable |
| SDC1 | SDC1^syndecan 1 | up-regulated | decreased | increased | variable | variable |
| TCF4 | TCF4^transcription factor 4 | up-regulated | decreased | increased | variable | variable |
| SRPX2 | SRPX2^sushi-repeat-containing protein, X-linked 2 | up-regulated | decreased | increased | variable | variable |
| TMEPAI | TMEPAI^transmembrane, prostate androgen induced RNA | up-regulated | decreased | increased | variable | variable |
| FBXO32 | FBXO32^F-box protein 32 | up-regulated | decreased | increased | variable | variable |
| CALB2 | CALB2^calbindin 2, 29kDa (calretinin) | up-regulated | decreased | increased | variable | variable |
| C21orf25 | C21orf25^chromosome 21 open reading frame 25 | up-regulated | decreased | increased | variable | variable |
| EFHD1 | EFHD1^EF-hand domain family, member D1 | up-regulated | decreased | increased | variable | variable |
| TIMP3 | TIMP3^TIMP metallopeptidase inhibitor 3 (Sorsby fundus dystrophy, pseudoinflammatory) | up-regulated | decreased | increased | variable | variable |
| DLC1 | DLC1^deleted in liver cancer 1 | up-regulated | decreased | increased | variable | variable |
| CDKN2B | CDKN2B^cyclin-dependent kinase inhibitor 2B (p15, inhibits CDK4) | up-regulated | decreased | increased | variable | variable |
| SNCAIP | SNCAIP^synuclein, alpha interacting protein (synphilin) | up-regulated | decreased | increased | variable | variable |
| KRT7 | KRT7^keratin 7 | up-regulated | decreased | increased | variable | variable |
| ACPP | ACPP^acid phosphatase, prostate | up-regulated | decreased | increased | variable | variable |
| SPON1 | SPON1^spondin 1, extracellular matrix protein | up-regulated | decreased | increased | variable | variable |
| POSTN | POSTN^periostin, osteoblast specific factor | up-regulated | decreased | increased | variable | variable |
| ASPN | ASPN^asporin | up-regulated | decreased | increased | variable | variable |
| C9orf19 | C9orf19^chromosome 9 open reading frame 19 | up-regulated | decreased | increased | variable | variable |
| PLXDC2 | PLXDC2^plexin domain containing 2 | up-regulated | decreased | increased | variable | variable |
| COL8A2 | COL8A2^collagen, type VIII, alpha 2 | up-regulated | decreased | increased | variable | variable |
| LMCD1 | LMCD1^LIM and cysteine-rich domains 1 | up-regulated | decreased | increased | variable | variable |
| NOX4 | NOX4^NADPH oxidase 4 | up-regulated | decreased | increased | variable | variable |
| WISP1 | WISP1^WNT1 inducible signaling pathway protein 1 | up-regulated | decreased | increased | variable | variable |
| COL11A1 | COL11A1^collagen, type XI, alpha 1 | up-regulated | decreased | increased | variable | variable |
| COL10A1 | COL10A1^collagen, type X, alpha 1(Schmid metaphyseal chondrodysplasia) | up-regulated | decreased | increased | variable | variable |
| ARHGDIB | ARHGDIB^Rho GDP dissociation inhibitor (GDI) beta | up-regulated | decreased | increased | variable | variable |
| GLIPR1 | GLIPR1^GLI pathogenesis-related 1 (glioma) | up-regulated | decreased | increased | variable | variable |
| KIAA1913 | KIAA1913^KIAA1913 | up-regulated | decreased | increased | variable | variable |
| SPOCD1 | SPOCD1^SPOC domain containing 1 | up-regulated | decreased | increased | variable | variable |
| F2R | F2R^coagulation factor II (thrombin) receptor | up-regulated | decreased | increased | variable | variable |
| LOH3CR2A | LOH3CR2A^loss of heterozygosity, 3, chromosomal region 2, gene A | up-regulated | decreased | increased | variable | variable |
| FLRT2 | FLRT2^fibronectin leucine rich transmembrane protein 2 | up-regulated | decreased | increased | variable | variable |
| SEMA3C | SEMA3C^sema domain, immunoglobulin domain (Ig), short basic domain, secreted, (semaphorin) 3C | up-regulated |  |  |  |  |
| TNFAIP6 | TNFAIP6^tumor necrosis factor, alpha-induced protein 6 | up-regulated |  |  |  |  |
| CSRP1 | CSRP1^cysteine and glycine-rich protein 1 | up-regulated |  |  |  |  |
| RAMP1 | RAMP1^receptor (G protein-coupled) activity modifying protein 1 | up-regulated |  |  |  |  |
| CNN1 | CNN1^calponin 1, basic, smooth muscle | up-regulated |  |  |  |  |
| ACTG2 | ACTG2^actin, gamma 2, smooth muscle, enteric | up-regulated |  |  |  |  |
| DMN | DMN^desmuslin | up-regulated |  |  |  |  |
| PPP1R14A | PPP1R14A^protein phosphatase 1, regulatory (inhibitor) subunit 14A | up-regulated |  |  |  |  |
| BGN | BGN^biglycan | up-regulated |  |  |  |  |
| EXTL1 | EXTL1^exostoses (multiple)-like 1 | up-regulated |  |  |  |  |
| SCD | SCD^stearoyl-CoA desaturase (delta-9-desaturase) | up-regulated |  |  |  |  |
| GPAM | GPAM^glycerol-3-phosphate acyltransferase, mitochondrial | up-regulated |  |  |  |  |
| COL4A2 | COL4A2^collagen, type IV, alpha 2 | up-regulated |  |  |  |  |
| COMP | COMP^cartilage oligomeric matrix protein | up-regulated |  |  |  |  |
| PRICKLE1 | PRICKLE1^prickle homolog 1 (Drosophila) | up-regulated |  |  |  |  |
| FZD8 | FZD8^frizzled homolog 8 (Drosophila) | up-regulated |  |  |  |  |
| SERPINB6 | SERPINB6^serpin peptidase inhibitor, clade B (ovalbumin), member 6 | up-regulated |  |  |  |  |
| KHDRBS3 | KHDRBS3^KH domain containing, RNA binding, signal transduction associated 3 | up-regulated |  |  |  |  |
| ITGA11 | ITGA11^integrin, alpha 11 | up-regulated |  |  |  |  |
| CPE | CPE^carboxypeptidase E | up-regulated |  |  |  |  |
| MRPS6 | MRPS6^mitochondrial ribosomal protein S6 | up-regulated |  |  |  |  |
| IVNS1ABP | IVNS1ABP^influenza virus NS1A binding protein | up-regulated |  |  |  |  |
| MT1F | MT1F^metallothionein 1F | up-regulated |  |  |  |  |
| IL6 | IL6^interleukin 6 (interferon, beta 2) | up-regulated |  |  |  |  |
| FJX1 | FJX1^four jointed box 1 (Drosophila) | up-regulated |  |  |  |  |
| NDRG1 | NDRG1^N-myc downstream regulated gene 1 | up-regulated |  |  |  |  |
| BAALC | BAALC^brain and acute leukemia, cytoplasmic | up-regulated |  |  |  |  |
| ID1 | ID1^inhibitor of DNA binding 1, dominant negative helix-loop-helix protein | up-regulated |  |  |  |  |
| ID3 | ID3^inhibitor of DNA binding 3, dominant negative helix-loop-helix protein | up-regulated |  |  |  |  |
| MEOX1 | MEOX1^mesenchyme homeobox 1 | up-regulated |  |  |  |  |
| SLC25A19 | SLC25A19^solute carrier family 25 (mitochondrial thiamine pyrophosphate carrier), member 19 | up-regulated |  |  |  |  |
| STS-1 | STS-1^Cbl-interacting protein Sts-1 | up-regulated |  |  |  |  |
| GADD45B | GADD45B^growth arrest and DNA-damage-inducible, beta | up-regulated |  |  |  |  |
| F2RL1 | F2RL1^coagulation factor II (thrombin) receptor-like 1 | up-regulated |  |  |  |  |
| C21orf7 | C21orf7^chromosome 21 open reading frame 7 | up-regulated |  |  |  |  |
| HNRPAB | HNRPAB^heterogeneous nuclear ribonucleoprotein A/B | up-regulated |  |  |  |  |
| FNTB | FNTB^farnesyltransferase, CAAX box, beta | up-regulated |  |  |  |  |
| SOX9 | SOX9^SRY (sex determining region Y)-box 9 (campomelic dysplasia, autosomal sex-reversal) | up-regulated |  |  |  |  |
| ATP1B1 | ATP1B1^ATPase, Na+/K+ transporting, beta 1 polypeptide | up-regulated |  |  |  |  |
| LARGE | LARGE^like-glycosyltransferase | up-regulated |  |  |  |  |
| MICAL2 | MICAL2^microtubule associated monoxygenase, calponin and LIM domain containing 2 | up-regulated |  |  |  |  |
| NET1 | NET1^neuroepithelial cell transforming gene 1 | up-regulated |  |  |  |  |
| MAP3K4 | MAP3K4^mitogen-activated protein kinase kinase kinase 4 | up-regulated |  |  |  |  |
| BHLHB2 | BHLHB2^basic helix-loop-helix domain containing, class B, 2 | up-regulated |  |  |  |  |
| DNAJB9 | DNAJB9^DnaJ (Hsp40) homolog, subfamily B, member 9 | up-regulated |  |  |  |  |
| ZNF295 | ZNF295^zinc finger protein 295 | up-regulated |  |  |  |  |
| NP | NP^nucleoside phosphorylase | up-regulated |  |  |  |  |
| IL11 | IL11^interleukin 11 | up-regulated |  |  |  |  |
| KCNG1 | KCNG1^potassium voltage-gated channel, subfamily G, member 1 | up-regulated |  |  |  |  |
| ADAM19 | ADAM19^ADAM metallopeptidase domain 19 (meltrin beta) | up-regulated |  |  |  |  |
| PRPS1 | PRPS1^phosphoribosyl pyrophosphate synthetase 1 | up-regulated |  |  |  |  |
| HS3ST3A1 | HS3ST3A1^heparan sulfate (glucosamine) 3-O-sulfotransferase 3A1 | up-regulated |  |  |  |  |
| UCK2 | UCK2^uridine-cytidine kinase 2 | up-regulated |  |  |  |  |
| POLE3 | POLE3^polymerase (DNA directed), epsilon 3 (p17 subunit) | up-regulated |  |  |  |  |
| SERPINE1 | SERPINE1^serpin peptidase inhibitor, clade E (nexin, plasminogen activator inhibitor type 1), member 1 | up-regulated |  |  |  |  |
| TPM1 | TPM1^tropomyosin 1 (alpha) | up-regulated |  |  |  |  |
| RAI14 | RAI14^retinoic acid induced 14 | up-regulated |  |  |  |  |
| WSB2 | WSB2^WD repeat and SOCS box-containing 2 | up-regulated |  |  |  |  |
| ARMET | ARMET^arginine-rich, mutated in early stage tumors | up-regulated |  |  |  |  |
| CHN1 | CHN1^chimerin (chimaerin) 1 | up-regulated |  |  |  |  |
| FN1 | FN1^fibronectin 1 | up-regulated |  |  |  |  |
| APAF1 | APAF1^apoptotic peptidase activating factor 1 | up-regulated |  |  |  |  |
| PAWR | PAWR^PRKC, apoptosis, WT1, regulator | up-regulated |  |  |  |  |
| PFKP | PFKP^phosphofructokinase, platelet | up-regulated |  |  |  |  |
| CDH2 | CDH2^cadherin 2, type 1, N-cadherin (neuronal) | up-regulated |  |  |  |  |
| COL4A1 | COL4A1^collagen, type IV, alpha 1 | up-regulated |  |  |  |  |
| LTBP2 | LTBP2^latent transforming growth factor beta binding protein 2 | up-regulated |  |  |  |  |
| DACT1 | DACT1^dapper, antagonist of beta-catenin, homolog 1 (Xenopus laevis) | up-regulated |  |  |  |  |
| P4HA2 | P4HA2^procollagen-proline, 2-oxoglutarate 4-dioxygenase (proline 4-hydroxylase), alpha polypeptide II | up-regulated |  |  |  |  |
| INHBE | INHBE^inhibin, beta E | up-regulated |  |  |  |  |
| LIMS1 | LIMS1^LIM and senescent cell antigen-like domains 1 | up-regulated |  |  |  |  |
| GLS | GLS^glutaminase | up-regulated |  |  |  |  |
| HNT | HNT^neurotrimin | up-regulated |  |  |  |  |
| TMEM2 | TMEM2^transmembrane protein 2 | up-regulated |  |  |  |  |
| LMO4 | LMO4^LIM domain only 4 | up-regulated |  |  |  |  |
| CREB3L2 | CREB3L2^cAMP responsive element binding protein 3-like 2 | up-regulated |  |  |  |  |
| SLC39A14 | SLC39A14^solute carrier family 39 (zinc transporter), member 14 | up-regulated |  |  |  |  |
| CNN3 | CNN3^calponin 3, acidic | up-regulated |  |  |  |  |
| KIAA0746 | KIAA0746^KIAA0746 protein | up-regulated |  |  |  |  |
| C7orf24 | C7orf24^chromosome 7 open reading frame 24 | up-regulated |  |  |  |  |
| H2AFZ | H2AFZ^H2A histone family, member Z | up-regulated |  |  |  |  |
| STMN1 | STMN1^stathmin 1/oncoprotein 18 | up-regulated |  |  |  |  |
| TK1 | TK1^thymidine kinase 1, soluble | up-regulated |  |  |  |  |
| PTTG1 | PTTG1^pituitary tumor-transforming 1 | up-regulated |  |  |  |  |
| TDG | TDG^thymine-DNA glycosylase | up-regulated |  |  |  |  |
| KIF21A | KIF21A^kinesin family member 21A | up-regulated |  |  |  |  |
| EXOSC9 | EXOSC9^exosome component 9 | up-regulated |  |  |  |  |
| RECQL | RECQL^RecQ protein-like (DNA helicase Q1-like) | up-regulated |  |  |  |  |
| RRM1 | RRM1^ribonucleotide reductase M1 polypeptide | up-regulated |  |  |  |  |
| CSE1L | CSE1L^CSE1 chromosome segregation 1-like (yeast) | up-regulated |  |  |  |  |
| C9orf46 | C9orf46^chromosome 9 open reading frame 46 | up-regulated |  |  |  |  |
| EXOSC8 | EXOSC8^exosome component 8 | up-regulated |  |  |  |  |
| LIN9 | LIN9^lin-9 homolog (C. elegans) | up-regulated |  |  |  |  |
| POLE2 | POLE2^polymerase (DNA directed), epsilon 2 (p59 subunit) | up-regulated |  |  |  |  |
| DNAJC9 | DNAJC9^DnaJ (Hsp40) homolog, subfamily C, member 9 | up-regulated |  |  |  |  |
| NEDD4 | NEDD4^neural precursor cell expressed, developmentally down-regulated 4 | up-regulated |  |  |  |  |
| DOCK10 | DOCK10^dedicator of cytokinesis 10 | up-regulated |  |  |  |  |
| MPP4 | MPP4^membrane protein, palmitoylated 4 (MAGUK p55 subfamily member 4) | up-regulated |  |  |  |  |
| AUTS2 | AUTS2^autism susceptibility candidate 2 | up-regulated | increased | decreased | decreased | variable |
| COL7A1 | COL7A1^collagen, type VII, alpha 1 (epidermolysis bullosa, dystrophic, dominant and recessive) | up-regulated | increased | decreased | decreased | variable |
| IER3 | IER3^immediate early response 3 | up-regulated | increased | decreased | decreased | variable |
| FIBCD1 | FIBCD1^fibrinogen C domain containing 1 | up-regulated | increased | decreased | decreased | variable |
| FSTL3 | FSTL3^follistatin-like 3 (secreted glycoprotein) | up-regulated | increased | decreased | decreased | variable |
| SDF2L1 | SDF2L1^stromal cell-derived factor 2-like 1 | up-regulated | increased | decreased | decreased | variable |
| TNFRSF12A | TNFRSF12A^tumor necrosis factor receptor superfamily, member 12A | up-regulated | increased | decreased | decreased | variable |
| PDGFC | PDGFC^platelet derived growth factor C | up-regulated | increased | decreased | decreased | variable |
| NAP1L3 | NAP1L3^nucleosome assembly protein 1-like 3 | up-regulated | increased | decreased | decreased | variable |
| PLOD2 | PLOD2^procollagen-lysine, 2-oxoglutarate 5-dioxygenase 2 | up-regulated | increased | decreased | decreased | variable |
| ENPP1 | ENPP1^ectonucleotide pyrophosphatase/phosphodiesterase 1 | up-regulated | increased | decreased | decreased | variable |
| SPP1 | SPP1^secreted phosphoprotein 1 (osteopontin, bone sialoprotein I, early T-lymphocyte activation 1) | up-regulated | increased | decreased | decreased | variable |
| ADAMTS4 | ADAMTS4^ADAM metallopeptidase with thrombospondin type 1 motif, 4 | up-regulated | increased | decreased | decreased | variable |
| SLC20A1 | SLC20A1^solute carrier family 20 (phosphate transporter), member 1 | up-regulated | increased | decreased | decreased | variable |
| FKBP11 | FKBP11^FK506 binding protein 11, 19 kDa | up-regulated | increased | decreased | decreased | variable |
| SLC7A5 | SLC7A5^solute carrier family 7 (cationic amino acid transporter, y+ system), member 5 | up-regulated | increased | decreased | decreased | variable |
| MYO10 | MYO10^myosin X | up-regulated | increased | decreased | decreased | variable |
| MLPH | MLPH^melanophilin | up-regulated | increased | decreased | decreased | variable |
| PDXP | PDXP^pyridoxal (pyridoxine, vitamin B6) phosphatase | up-regulated | increased | decreased | decreased | variable |
| OXTR | OXTR^oxytocin receptor | up-regulated | increased | decreased | decreased | variable |
| ENC1 | ENC1^ectodermal-neural cortex (with BTB-like domain) | up-regulated | increased | decreased | decreased | variable |
| RGS4 | RGS4^regulator of G-protein signaling 4 | up-regulated | increased | decreased | decreased | variable |
| KRT18 | KRT18^keratin 18 | up-regulated | increased | decreased | decreased | variable |
| SPHK1 | SPHK1^sphingosine kinase 1 | up-regulated | increased | decreased | decreased | variable |
| MCM7 | MCM7^minichromosome maintenance complex component 7 | up-regulated | increased | decreased | decreased | variable |
| EVL | EVL^Enah/Vasp-like | up-regulated | increased | decreased | decreased | variable |
| MCM5 | MCM5^minichromosome maintenance complex component 5 | up-regulated | increased | decreased | decreased | variable |
| PSMD2 | PSMD2^proteasome (prosome, macropain) 26S subunit, non-ATPase, 2 | up-regulated | increased | decreased | decreased | variable |
| ETV4 | ETV4^ets variant gene 4 (E1A enhancer binding protein, E1AF) | up-regulated | increased | decreased | decreased | variable |
| TNFRSF10D | TNFRSF10D^tumor necrosis factor receptor superfamily, member 10d, decoy with truncated death domain | up-regulated | increased | decreased | decreased | variable |
| TRIP13 | TRIP13^thyroid hormone receptor interactor 13 | up-regulated | increased | decreased | decreased | variable |
| PFKFB4 | PFKFB4^6-phosphofructo-2-kinase/fructose-2,6-biphosphatase 4 | up-regulated | increased | decreased | decreased | variable |
| CDT1 | CDT1^chromatin licensing and DNA replication factor 1 | up-regulated | increased | decreased | decreased | variable |
| PRR6 | PRR6^proline rich 6 | up-regulated | increased | decreased | decreased | variable |
| UBE2S | UBE2S^ubiquitin-conjugating enzyme E2S | up-regulated | increased | decreased | decreased | variable |
| FEN1 | FEN1^flap structure-specific endonuclease 1 | up-regulated | increased | decreased | decreased | variable |
| DEPDC1 | DEPDC1^DEP domain containing 1 | up-regulated | increased | decreased | decreased | variable |
| TOP2A | TOP2A^topoisomerase (DNA) II alpha 170kDa | up-regulated | increased | decreased | decreased | variable |
| CTPS | CTPS^CTP synthase | up-regulated | increased | decreased | decreased | variable |
| NETO2 | NETO2^neuropilin (NRP) and tolloid (TLL)-like 2 | up-regulated | increased | decreased | decreased | variable |
| HAS2 | HAS2^hyaluronan synthase 2 | up-regulated | increased | decreased | decreased | variable |
| UAP1 | UAP1^UDP-N-acteylglucosamine pyrophosphorylase 1 | up-regulated | increased | decreased | decreased | variable |
| GMNN | GMNN^geminin, DNA replication inhibitor | up-regulated | increased | decreased | decreased | variable |
| UPP1 | UPP1^uridine phosphorylase 1 | up-regulated | increased | decreased | decreased | variable |
| GDF6 | GDF6^growth differentiation factor 6 | up-regulated | increased | decreased | decreased | variable |
| TMPO | TMPO^thymopoietin | up-regulated | increased | decreased | decreased | variable |
| PPIL5 | PPIL5^peptidylprolyl isomerase (cyclophilin)-like 5 | up-regulated | increased | decreased | decreased | variable |
| MCM10 | MCM10^minichromosome maintenance complex component 10 | up-regulated | increased | decreased | decreased | variable |
| KIF11 | KIF11^kinesin family member 11 | up-regulated | increased | decreased | decreased | variable |
| EXO1 | EXO1^exonuclease 1 | up-regulated | increased | decreased | decreased | variable |
| E2F7 | E2F7^E2F transcription factor 7 | up-regulated | increased | decreased | decreased | variable |
| TFPI2 | TFPI2^tissue factor pathway inhibitor 2 | up-regulated | increased | decreased | decreased | variable |
| RFC4 | RFC4^replication factor C (activator 1) 4, 37kDa | up-regulated | increased | decreased | decreased | variable |
| MAD2L1 | MAD2L1^MAD2 mitotic arrest deficient-like 1 (yeast) | up-regulated | increased | decreased | decreased | variable |
| UHRF1 | UHRF1^ubiquitin-like, containing PHD and RING finger domains, 1 | up-regulated | increased | decreased | decreased | variable |
| CDCA5 | CDCA5^cell division cycle associated 5 | up-regulated | increased | decreased | decreased | variable |
| KIF20A | KIF20A^kinesin family member 20A | up-regulated | increased | decreased | decreased | variable |
| TTK | TTK^TTK protein kinase | up-regulated | increased | decreased | decreased | variable |
| CDCA7 | CDCA7^cell division cycle associated 7 | up-regulated | increased | decreased | decreased | variable |
| ASPM | ASPM^asp (abnormal spindle) homolog, microcephaly associated (Drosophila) | up-regulated | increased | decreased | decreased | variable |
| DLG7 | DLG7^discs, large homolog 7 (Drosophila) | up-regulated | increased | decreased | decreased | variable |
| MELK | MELK^maternal embryonic leucine zipper kinase | up-regulated | increased | decreased | decreased | variable |
| SPAG5 | SPAG5^sperm associated antigen 5 | up-regulated | increased | decreased | decreased | variable |
| ANLN | ANLN^anillin, actin binding protein | up-regulated | increased | decreased | decreased | variable |
| CDC2 | CDC2^cell division cycle 2, G1 to S and G2 to M | up-regulated | increased | decreased | decreased | variable |
| FBXO5 | FBXO5^F-box protein 5 | up-regulated | increased | decreased | decreased | variable |
| CENPA | CENPA^centromere protein A | up-regulated | increased | decreased | decreased | variable |
| CCNA2 | CCNA2^cyclin A2 | up-regulated | increased | decreased | decreased | variable |
| ATAD2 | ATAD2^ATPase family, AAA domain containing 2 | up-regulated | increased | decreased | decreased | variable |
| BUB1 | BUB1^BUB1 budding uninhibited by benzimidazoles 1 homolog (yeast) | up-regulated | increased | decreased | decreased | variable |
| CDCA8 | CDCA8^cell division cycle associated 8 | up-regulated | increased | decreased | decreased | variable |
| NUSAP1 | NUSAP1^nucleolar and spindle associated protein 1 | up-regulated | increased | decreased | decreased | variable |
| RAD51AP1 | RAD51AP1^RAD51 associated protein 1 | up-regulated | increased | decreased | decreased | variable |
| KIAA0101 | KIAA0101^KIAA0101 | up-regulated | increased | decreased | decreased | variable |
| CCNB2 | CCNB2^cyclin B2 | up-regulated | increased | decreased | decreased | variable |
| UBE2C | UBE2C^ubiquitin-conjugating enzyme E2C | up-regulated | increased | decreased | decreased | variable |
| CENPE | CENPE^centromere protein E, 312kDa | up-regulated | increased | decreased | decreased | variable |
| CDC20 | CDC20^cell division cycle 20 homolog (S. cerevisiae) | up-regulated | increased | decreased | decreased | variable |
| KIF2C | KIF2C^kinesin family member 2C | up-regulated | increased | decreased | decreased | variable |
| PRC1 | PRC1^protein regulator of cytokinesis 1 | up-regulated | increased | decreased | decreased | variable |
| RFC5 | RFC5^replication factor C (activator 1) 5, 36.5kDa | up-regulated | increased | decreased | decreased | variable |
| CCNB1 | CCNB1^cyclin B1 | up-regulated | increased | decreased | decreased | variable |
| CKS1B | CKS1B^CDC28 protein kinase regulatory subunit 1B | up-regulated | increased | decreased | decreased | variable |
| CKAP2 | CKAP2^cytoskeleton associated protein 2 | up-regulated | increased | decreased | decreased | variable |
| AURKB | AURKB^aurora kinase B | up-regulated | increased | decreased | decreased | variable |
| LPXN | LPXN^leupaxin | up-regulated | increased | decreased | decreased | variable |
| SAMD11 | SAMD11^sterile alpha motif domain containing 11 | up-regulated | increased | decreased | decreased | variable |
| SMYD3 | SMYD3^SET and MYND domain containing 3 | up-regulated | increased | decreased | decreased | variable |
| CDK2 | CDK2^cyclin-dependent kinase 2 | up-regulated | increased | decreased | decreased | variable |
| MTP18 | MTP18^mitochondrial protein 18 kDa | up-regulated | increased | decreased | decreased | variable |
| CDCA3 | CDCA3^cell division cycle associated 3 | up-regulated | increased | decreased | decreased | variable |
| MCM4 | MCM4^minichromosome maintenance complex component 4 | up-regulated | increased | decreased | decreased | variable |
| TACC3 | TACC3^transforming, acidic coiled-coil containing protein 3 | up-regulated | increased | decreased | decreased | variable |
| TPX2 | TPX2^TPX2, microtubule-associated, homolog (Xenopus laevis) | up-regulated | increased | decreased | decreased | variable |
| GTSE1 | GTSE1^G-2 and S-phase expressed 1 | up-regulated | increased | decreased | decreased | variable |
| POLA2 | POLA2^polymerase (DNA directed), alpha 2 (70kD subunit) | up-regulated | increased | decreased | decreased | variable |
| KIAA0907 | KIAA0907^KIAA0907 | up-regulated | increased | decreased | decreased | variable |
| TGM2 | TGM2^transglutaminase 2 (C polypeptide, protein-glutamine-gamma-glutamyltransferase) | up-regulated | increased | decreased | decreased | variable |
| MPHOSPH1 | MPHOSPH1^M-phase phosphoprotein 1 | up-regulated | increased | decreased | decreased | variable |
| PLK4 | PLK4^polo-like kinase 4 (Drosophila) | up-regulated | increased | decreased | decreased | variable |
| ANKRD1 | ANKRD1^ankyrin repeat domain 1 (cardiac muscle) | up-regulated | increased | decreased | decreased | variable |
| NKX3-1 | NKX3-1^NK3 homeobox 1 | up-regulated | increased | decreased | decreased | variable |
| IGFBP3 | IGFBP3^insulin-like growth factor binding protein 3 | up-regulated | increased | decreased | decreased | variable |
| CRLF1 | CRLF1^cytokine receptor-like factor 1 | up-regulated | increased | decreased | decreased | variable |
| M-RIP | M-RIP^myosin phosphatase-Rho interacting protein | up-regulated | increased | decreased | decreased | variable |
| TP53I3 | TP53I3^tumor protein p53 inducible protein 3 | up-regulated | increased | decreased | decreased | variable |
| NES | NES^nestin | up-regulated | increased | decreased | decreased | variable |
| CDK5RAP2 | CDK5RAP2^CDK5 regulatory subunit associated protein 2 | up-regulated | increased | decreased | decreased | variable |
| RNASEH2A | RNASEH2A^ribonuclease H2, subunit A | up-regulated | increased | decreased | decreased | variable |
| ITGA3 | ITGA3^integrin, alpha 3 (antigen CD49C, alpha 3 subunit of VLA-3 receptor) | up-regulated | increased | decreased | decreased | variable |
| PTPRN | PTPRN^protein tyrosine phosphatase, receptor type, N | up-regulated | increased | decreased | decreased | variable |
|  |  |  |  |  |  |  |
