# Supplementary material for: Egr-1 Induces a Profibrotic Injury/Repair Gene Program Associated with Systemic Sclerosis
Source: PLoS One. 2011 Sep 13;6(9):e23082. doi: 10.1371/journal.pone.0023082 (PMC3172216; doi:10.1371/journal.pone.0023082)
Supplement: Table S2 — Top gene information of Egr1m48-C48 and TGF-β 48-C48 (p-value<0.001) and fold-change larger than two. (DOCX) [file pone.0023082.s003.docx]

**Table S2**
